# Topographical Body Fat Distribution Links to Amino Acid and Lipid Metabolism in Healthy Non-Obese Women

**DOI:** 10.1371/journal.pone.0073445

**Published:** 2013-09-11

**Authors:** Francois-Pierre J. Martin, Ivan Montoliu, Sebastiano Collino, Max Scherer, Philippe Guy, Isabelle Tavazzi, Anita Thorimbert, Sofia Moco, Megan P. Rothney, David L. Ergun, Maurice Beaumont, Fiona Ginty, Salah D. Qanadli, Lucie Favre, Vittorio Giusti, Serge Rezzi

**Affiliations:** 1 Metabolomics and Biomarkers, Nestec Ltd., Nestle Research Center, Lausanne, Switzerland; 2 Applied Mathematics, Nestec Ltd., Nestle Research Center, Lausanne, Switzerland; 3 Diagnostics and Biomedical Technology Organization, GE Global Research Center, Niskayuna, New York, United States of America; 4 GE Healthcare, Madison, Wisconsin, United States of America; 5 Clinical Development Unit, Nestec Ltd., Nestle Research Center, Lausanne, Switzerland; 6 Cardiothoracic and Vascular Unit, Department of Radiology, University Hospital of Lausanne, Lausanne, Switzerland; 7 Service of Endocrinology, Diabetology and Metabolism, Department of Medicine, University Hospital of Lausanne, Lausanne, Switzerland; University of Bari & Consorzio Mario Negri Sud, Italy

## Abstract

Visceral adiposity is increasingly recognized as a key condition for the development of obesity related disorders, with the ratio between visceral adipose tissue (VAT) and subcutaneous adipose tissue (SAT) reported as the best correlate of cardiometabolic risk. In this study, using a cohort of 40 obese females (age: 25–45 y, BMI: 28–40 kg/m^2^) under healthy clinical conditions and monitored over a 2 weeks period we examined the relationships between different body composition parameters, estimates of visceral adiposity and blood/urine metabolic profiles. Metabonomics and lipidomics analysis of blood plasma and urine were employed in combination with *in vivo* quantitation of body composition and abdominal fat distribution using iDXA and computerized tomography. Of the various visceral fat estimates, VAT/SAT and VAT/total abdominal fat ratios exhibited significant associations with regio-specific body lean and fat composition. The integration of these visceral fat estimates with metabolic profiles of blood and urine described a distinct amino acid, diacyl and ether phospholipid phenotype in women with higher visceral fat. Metabolites important in predicting visceral fat adiposity as assessed by Random forest analysis highlighted 7 most robust markers, including tyrosine, glutamine, PC-*O* 44∶6, PC-*O* 44∶4, PC-*O* 42∶4, PC-*O* 40∶4, and PC-*O* 40∶3 lipid species. Unexpectedly, the visceral fat associated inflammatory profiles were shown to be highly influenced by inter-days and between-subject variations. Nevertheless, the visceral fat associated amino acid and lipid signature is proposed to be further validated for future patient stratification and cardiometabolic health diagnostics.

## Introduction

Overweight and obesity pandemic has made the discovery of their associated genome and metabolome one of the greatest public health challenges [1], [2]. Obesity, in particular visceral adiposity, associates with inflammation and insulin resistance which ultimately link to risks of type 2 diabetes and cardiometabolic disorders. The mechanisms leading to obesity related diseases (ORD) remain yet undefined but may involve platelet and vascular impairments [3], [4]. Moreover, visceral adiposity, also named central adiposity or obesity, is increasingly recognized as a key condition for the development of ORD [5]–[10].

The etiology and individual predisposition to visceral adiposity remain unclear. Over the last decades, both genetic and environmental promoters were investigated [11], [12], including genes and transcription factors associated with fat storage and obesity [12]–[16], genetic inheritability [17] and gut microbiota influence [18]. Nevertheless, similar obesogenic and diabetogenic conditions do not necessarily lead to a universal response to adiposity-associated cardiometabolic risks [19], [20]. Indeed, individuals with normal weight (body mass index, BMI<25) can express cardiometabolic abnormalities [19] putatively due to differences in body composition with a key role of sub-cutaneous versus visceral fat distribution.

From a diagnostic view, various indexes of visceral adiposity, combining anthropometric measures were proposed, including waist to hip ratio, (e.g. >0.9 for men and >0.85 for women) [21]. However, anthropometric-based stratification is prone to measurement errors. They also show limitations when applied across different populations (children versus adults) and ethnicities as universal health standards cutoffs. Moreover, these tools are insensitive to discerning the different sub-conditions of visceral adiposity and their associated metabolic risk factors. visceral adiposity includes indeed a complex topographical fat deposition, namely mesenteric and epicardial adipose tissues, and peripheric depots around organs like stomach, liver and kidneys, which associate with metabolic homeostatic loss [22]. The complex visceral fat compartment exhibits, beyond its role in dietary fat storage, signaling metabolic functions that interplay with endocrine and immune systems [12]. Modern imaging technologies based on magnetic resonance (MRI) and computed tomography (CT) can generate accurate regio-specific quantification of visceral fat depots. Nevertheless, it is unlikely that these imaging platforms can become universal tools for population screening and monitoring due to cost and access limitations.

In such a context, the identification of minimally-invasive, fast and reliable biomarkers to be used for effective and individual therapeutic solutions for visceral adiposity management and monitoring has become a key milestone to address the burden of ORD with cost effective diagnostics at epidemiological level. The development of such biomarkers may benefit from metabonomics, which is well suited to delineate metabolic phenotype, encapsulating the influence of various environment, drugs, dietary, lifestyle, genetics, and microbiome factors [23]–[25]. Recent applications have demonstrated the feasibility of associating specific metabolite profiles to body fat distribution [26], [27], and it is envisioned that metabonomics could deliver direct or indirect metabolic information that may generate new mechanistic knowledge of complex physiological processes.

We sought to identify visceral adiposity specific metabolic signature using a combination of holistic nuclear magnetic resonance (NMR) and targeted quantitative mass spectrometry (MS) analysis of urine and plasma from a well clinically characterized cohort of 40 healthy obese women. Subject characterization was based on clinical chemistry and body imaging including visceral adipose tissue (VAT) and subcutaneous adipose tissue (SAT) quantitation using gold standard CT completed with whole body Dual energy X-ray Absorptiometry (DXA) [28] scans. The VAT/SAT and VAT/total abdominal fat ratios, considered as cardiometabolic health indicators, were calculated to probe for metabolic-phenotype correlations [29]. In the present study, we assess how topography of visceral adiposity links to body composition parameters and metabolic status of healthy obese women.

## Materials and Methods

### Ethics Statement

This work was approved by the Ethical Committee of Lausanne University School Medicine (Lausanne, Switzerland). All participants gave written informed consent in French or in English as described in the consent procedure of the study protocol approved by the Ethics committee. The clinical study is registered at ClinicalTrials.gov with the identifier NCT01726647.

### Participants and Experimental Design

The observational study was conducted on 40 healthy obese Caucasian women at the out-patient obesity clinic of the University Hospital of Lausanne (CHUV), Switzerland. The participants had a BMI between 28 and 40, aged between 25 and 45 years old, showed no metabolic disease traits, and gave written informed consent. Additional exclusion criteria were diabetes, pregnancy, antibiotic therapy within 1 month prior to the beginning of the study, any therapy (contraception apart) within the run-in period of one week before the visit day, and eating disorders. In the current cohort no subjects suffered from hypertension, glucose intolerance, polycystic ovary syndrome, thyroid dysfunction and adrenal disorders. Subjects having recently undergone a weight loss of more than 3 kilos during the last 3 months were also excluded. During the conduction of the study, participants were asked to limit consumption of special foods (spices, supplements, etc.) for one week before the first visit day and to record their daily food intake. Fourteen subjects out of the 40 were taking contraception, and the subjects followed a randomized distribution across the spectrum of visceral fat adiposity. All the subjects showed normal menstrual cycle and took part in the study during the follicular phase of their menstrual cycle to minimize the confounding effects related to the hormonal changes. At a baseline (V0), subjects underwent a standard medical visit, where overnight fasting blood plasma samples were collected. One week later (V1), body composition was measured using CT (at CHUV) and iDXA (at the metabolic unit, Nestlé Research Center, Lausanne, Switzerland). Twenty-four-hours urine samples were collected, resting energy expenditure was measured by indirect calorimetry and a standard Oral Glucose Tolerance Test (OGTT, 75 g glucose) was conducted at V1. Glucose and insulin concentrations were measured at −15 min (t0), +30 min (t30), +60 min (t60), +90 min (t90), +120 min (t120) after glucose intake. One week later (V2), the subjects were asked to consume a standardized lunch and dinner to smooth inter-individual differences due to differences in diets. At this time point, overnight fasting plasma and 24-hours urine samples were collected, and fasting plasma samples again on the next day (V3).

### Anthropometric Measurements

Body weight was measured with a Detecto® scale with a precision of 0.2 kg; height was measured with a stadiometer with a precision of 0.5 cm. Body mass index (BMI) was calculated as weight/height squared (kg/m2). Waist and hip circumferences were taken respectively at the smallest standing horizontal circumference between the ribs and the iliac crest and in a horizontal plane around the maximum circumference of the buttocks, using a TEC anthropometric tape (Rollfix, Hoechstmass, Germany). The measurement was made to the nearest 0.1 cm at normal expiration. Three measurements were taken with the criterion that difference between the measurements had to be less than 2 cm. Additional measurements were taken when needed until this criterion was fulfilled.

### Body Composition Assessment and Energy Expenditure Assessments

Full body scan was performed to determine both abdominal fat distribution and total body composition. Total body scans were made on a GE Lunar iDXA system (software version: enCORE version 12.10.113) with scan mode automatically determined by the device and used the previously reported procedure [28]. For the DXA measurement, all subjects were wearing a hospital gown and had all metal artefacts removed. The iDXA unit was calibrated daily using the GE Lunar calibration phantom. A trained operator performed all scans following the operator’s manual for patient positioning and data acquisition. During the one-hour appointment, total body scans of each subject were performed twice with repositioning between scans. Scans were analyzed with the enCORE software (version 14.00.207). The ROIs were automatically determined by the enCORE software (Auto ROI) for total body, arms, legs, trunk, android, and gynoid regions. An experienced DXA operator also verified and, when indicated, repositioned the ROI placements (Expert ROI). In addition to iDXA scan, waist and hip measurements were performed.

The CT scan measures of the abdominal region, for the quantification of intra-peritoneal and sub-cutaneous fat, were performed on 64 multi-detector CT scanner (VCT Lightspeed, GE Medical Systems, Milwaukee, USA). Subjects lied in the supine position with their arms above their head and legs elevated with a cushion. A single scan (10 mm) of the abdomen is acquired at the level of L4–L5 vertebrae and analyzed for a cross-sectional area of adipose tissue, expressed in square centimeters. The following acquisition parameters were used: 120 Kv, 100–200 mA with z-axis dose modulation and a field of view 500 mm. Axial transverse images of 5 mm slice thickness are reconstructed using a standard kernel. The quantification process uses a semi interactive commercially available algorithm for segmentation of subcutaneous and intra-abdominal fat on the Advantage Window workstation (GE Medical Systems). Resting metabolic rate was measured by using open-circuit indirect calorimetry, with a Deltatrak II (Datex Instruments).

### Blood Pressure and Clinical Chemistry

Blood pressure was measured in the lying position using a digital pressure monitor (HEM-907, Omron). Mean arterial blood pressure (MAP) was also calculated from systolic (SYS) and diastolic (DIA) arterial blood pressure: MAP = 1/3 SYS +2/3 DIA). Blood samples were collected in the morning after an overnight fast. Glucose (Ecoline 100 Merck, KgaA, Darmstadt, Germany), total cholesterol (Roche CHOD-PAP, Roche Molecular Biochemicals Systems, GmbH, Mannheim, Germany), high-density lipoprotein cholesterol (HDL-C plus, second generation, Roche Diagnostic GmbH, Mannheim, Germany) and triglycerides (TG GPO-PAP, Roche Diagnostic) were measured using an automatic Hitachi 917 Roche apparatus. Low-density lipoprotein cholesterol was then calculated by the Friedwald’s formula. Plasma insulin was assayed by specific radioimmunoassay (Aldatis Insulin, code 10624, Casalecchio di Reno, BO, Italy). Urea, creatinine, sodium and potassium concentrations, ALAT, ASAT, γGT, were measured by SSCC (Société Suisse de Chimie Clinique) 37°C method with kit Roche and kit BioMérieux with automatic Hitachi 917 Roche, according to routine analytic methods. Intra-assay precision error (CV) for these measurements was 1.0–3.1%. All biochemical analyses were carried out by certified laboratories (ISO/CEI 17025); they were run in duplicate or triplicate samples, after having been previously tested in a healthy population. Insulin resistance status was assessed as homeostasis model assessment of insulin resistance (HOMA-IR): insulin (µU/mL)×glucose (mmol/L)/22.5. Finally, free fatty acids were analyzed by WAKO NEFA-HR (300 µL plasma, EDTA) using Siemens XPAND DIMENSION (WA2 434–91795, WA2 436–91995, USA).

### Metabonomics Analysis

Targeted LC-MS/MS metabonomic approach using the Biocrates Life Sciences Absolute*IDQ*TM kit was applied to plasma samples as previously published [30]. Briefly, well plate preparation and sample application and extraction were carried out according to the manufacturer's instructions. A final volume of 10 µl of plasma was loaded onto the provided 96-well plate. Liquid chromatography was realized on a Dionex Ultimate 3000 ultra high pressure liquid chromatography (UHPLC) system (Dionex AG, Olten, Switzerland) coupled to a 3200 Q TRAP mass spectrometer (AB Sciex; Foster City, CA, USA) fitted with a TurboV ion source operating in electrospray ionization (ESI) mode. Sample extracts (20 µl) were injected twice (in positive and negative ESI modes) via direct infusion using a gradient flow rate of 0–2.4 min: 30 µl/min, 2.4–2.8 min: 200 µl/min, 2.9–3 min: 30 µl/min. MS source parameters were set at: desolvation temperature (TEM): 200°C, high voltage: −4500 V (ESI −), 5500 V (ESI +), curtain (CUR) and nebuliser (GS1 and GS2) gases: nitrogen; 20, 40, and 50 psi; respectively, nitrogen collision gas pressure: 5 mTorr. MS/MS acquisition was realised in scheduled reaction monitoring (SRM) mode with optimised declustering potential values for the 163 metabolites screened in the assay. Raw data files (Analyst software, version 1.5.1; AB Sciex, Foster City, CA, USA) were imported into the provided analysis software MetIQ to calculate metabolite concentrations. List of all detectable metabolites is available from Biocrates Life Sciences, Austria (http://biocrates.com). See Text S1 for more details. Samples were also subjected to analysis for inflammation markers quantification by UPLC-ESI-MS/MS using isotope dilution technique. See Text S2 for more details.

Heparinized blood plasma samples (400 µL) were introduced into 5 mm NMR tubes with 200 µL of deuterated phosphate buffer solution (KH_2_PO_4_ with a final concentration of 0.2 M). Deuterium was employed as locking substance. Twenty-four-hours urine samples (400 µL) were introduced into 5 mm NMR tubes with 200 µL of deuterated phosphate buffer solution (KH_2_PO_4_ with a final concentration of 0.2 M, and containing 1 mM of sodium 3-(trimethylsilyl)-[2,2,3,3-2H_4_]-1-propionate (TSP). Metabolic profiles were measured on a Bruker Avance III 600 MHz spectrometer equipped with an inverse 5 mm cryogenic probe at 300 K (Bruker Biospin, Rheinstetten, Germany). See Text S3 for more details.

### Chemometrics

Due to the non-normal distribution of the visceral adiposity, the following parameters were employed for the subsequent metabonomics analysis: log-transform value of the visceral fat content, of the intraperitoneal/subcutaneous fat ratio (ratio 1), or of the intraperitoneal/abdominal fat ratio (ratio 2). The plasma and urine NMR spectra were converted into 22 K data points over the range of δ 0.2–10.0 ppm using an in-house developed MATLAB routine excluding the water residue signal between δ4.68–5.10 ppm. Chemical shift intensities were normalized to the sum of all intensities within the specified range prior to chemometric analysis for urine and plasma samples. In addition, 24-hours urine spectral data were also normalized to creatinine values quantified in parallel using an adapted Jaffe method.

Chemometric analysis was performed using the software package SIMCA-P+ (version 12.0, Umetrics AB, Umeå, Sweden) and in-house developed MATLAB (The MathWorks Inc., Natick, MA, USA) routines. In order to detect the presence of similarities between metabolic profiles, Principal Component Analysis (PCA) [31], Projection to Latent Structure (PLS) [32], and the Orthogonal Projection to Latent Structures (O-PLS) [33] were used. Analyses were conducted to investigate relationships between body composition, OGTT response and metabolic profiles. Seven-fold cross validation was used to assess the validity of the model [34]. The classification accuracy of the O-PLS-DA model was established from the predicted samples in the 7-fold cross-validation cycle. The modeling was also tested for assessing inter-days metabolic variations, by considering each visit separately.

In addition, targeted MS data were analyzed by Random Forests (RF™) using the package ‘randomForest’ [35]. In particular, the selection of the most robust markers required additional multivariate data analyses using RF™ (with 500 trees, and a random variable sampling at tree split of 5) and the implemented variable importance features in RF™ (mean decreases in accuracy/node impurity) to determine variables that discriminates better subjects according to their visceral fat status (Q1 versus Q4) as assessed using either value of the intraperitoneal fat volume, ratio 1 and ratio 2.

Spearman autocorrelation matrices were calculated using R and corresponding graphs were produced using the package Rgraphviz v.1.32.0. Univariate significance tests for confirmation were also performed in R.

## Results

### Clinical Characteristics of the Obese Cohort

Anthropometric and clinical parameters of the cohort were measured at visit 2 (V2) and main parameters are shown as per stratification in four quartiles (Q1–4, n = 10) based on the log_10_ value of the intraperitoneal fat volume measured using CT (Table S1), log_10_ value of intraperitoneal/subcutaneous fat ratio (ratio 1, Table S2), or log_10_ value of intraperitoneal/abdominal fat ratio (ratio 2, Table 1).

**Table 1:** Descriptive statistics of subjects stratified according to intraperitoneal/abdominal fat ratio.

Factor	Q1	Q2	Q3	Q4	Mann-Whitney p value (Q1/Q4)
**Log10 Ratio2**	−**0.7±0.05**	−**0.61±0.02**	−**0.52±0.02**	−**0.40±0.06**	**<0.0001**
**IPVF, mL**	**3065.1±695.5**	**4223±837.6**	**5111.2±1036.3**	**5407±1564.6**	**0.0002**
**HOMA-IR**	**4.24±2.02**	**4.95±1.49**	**6.06±1.87**	**6.12±1.23**	**0.011**
**Insulin, (µU/mL)**	**18.6±9.21**	**22.12±6.32**	**24.36±7.22**	**25.44±4.62**	**0.014**
Glucose, mmol/L	4.95±0.35	5.17±0.52	5.41±0.49	5.37±0.5	0.057
TG, mmol/L	1.04±0.43	2.25±2.1	1.28±0.45	1.52±0.57	0.093
ALAT/ASAT ratio	0.86±0.25	0.91±0.21	0.99±0.3	1.08±0.34	0.120
Age, years	33.9±4.89	32.8±3.58	38±4.42	37.6±5.82	0.138
ALAT, U/L	18.4±6.11	19.2±5.07	23.5±8.34	27.1±13.28	0.139
HDL, mmol/L	1.54±0.43	1.32±0.29	1.38±0.25	1.32±0.24	0.181
GGT, U/L	20±11.86	17.5±6.88	21.1±4.84	25.44±11.26	0.191
Creatinine, mmol/L	65.6±9.45	65.2±11.2	64.78±9.28	70.3±6.53	0.256
HDL/Chol ratio	3.77±1.07	4.42±1.22	3.99±0.97	4.24±0.95	0.289
Na, mmol/L	140.4±1.35	140.8±1.32	141.5±1.58	139.9±1.1	0.328
Waist/Hip ratio	0.8±0.07	0.81±0.06	0.85±0.07	0.84±0.09	0.351
Urates, µmol/L	275.2±41.93	263.22±71.45	303.4±75.28	285±31.7	0.352
Calorimetry, kcal/24 h	1357±191.78	1434±142.61	1469±152.49	1433±210.82	0.363
ASAT, U/L	21.4±3.24	21.4±4.48	24±6.94	24.5±6.7	0.401
Waist, cm	97.28±8.28	103.39±8.7	108.72±11.71	104.73±13.84	0.458
Hip, cm	122±5.47	128±7.48	127.34±6.29	122.28±9.65	0.564
NEFAs, µmol/L	544.5±201.51	580.6±301.38	596.2±185.79	585.1±188.62	0.664
K, mmol/L	4.05±0.18	4.1±0.18	3.99±0.25	4.04±0.18	0.876
MAP, mmHg	57.8±18.6	71.1±19.75	62.4±20.97	57.8±14.15	0.879
Cholesterol, mmol/L	5.52±1.01	5.58±0.85	5.31±0.68	5.48±0.97	0.909
BMI, kg/m2	34.01±3.27	36.34±3.62	37±2.95	34.59±4.42	0.939
LDL, mmol/L	3.5±0.97	3.56±0.88	3.34±0.61	3.47±0.79	1

Key: Qi: data for population quartile i according to intraperitoneal/abdominal fat ratio. BMI = body mass index, HDL-C =  high density lipoprotein cholesterol, homeostasis model assessment of insulin resistance =  HOMA-IR, LDL-C =  low density lipoprotein cholesterol, TG =  triglycerides, MAP =  mean arterial blood pressure, ALAT =  alanine aminotransferase, ASAT =  aspartate aminotransferase, GGT =  gamma-glutamyl transpeptidase, NEFAs = non esterified fatty acids. Data are reported as mean values ± SD.

### Relationships between Visceral Fat Indicators and other Body Composition Parameters

**Figure 1:**
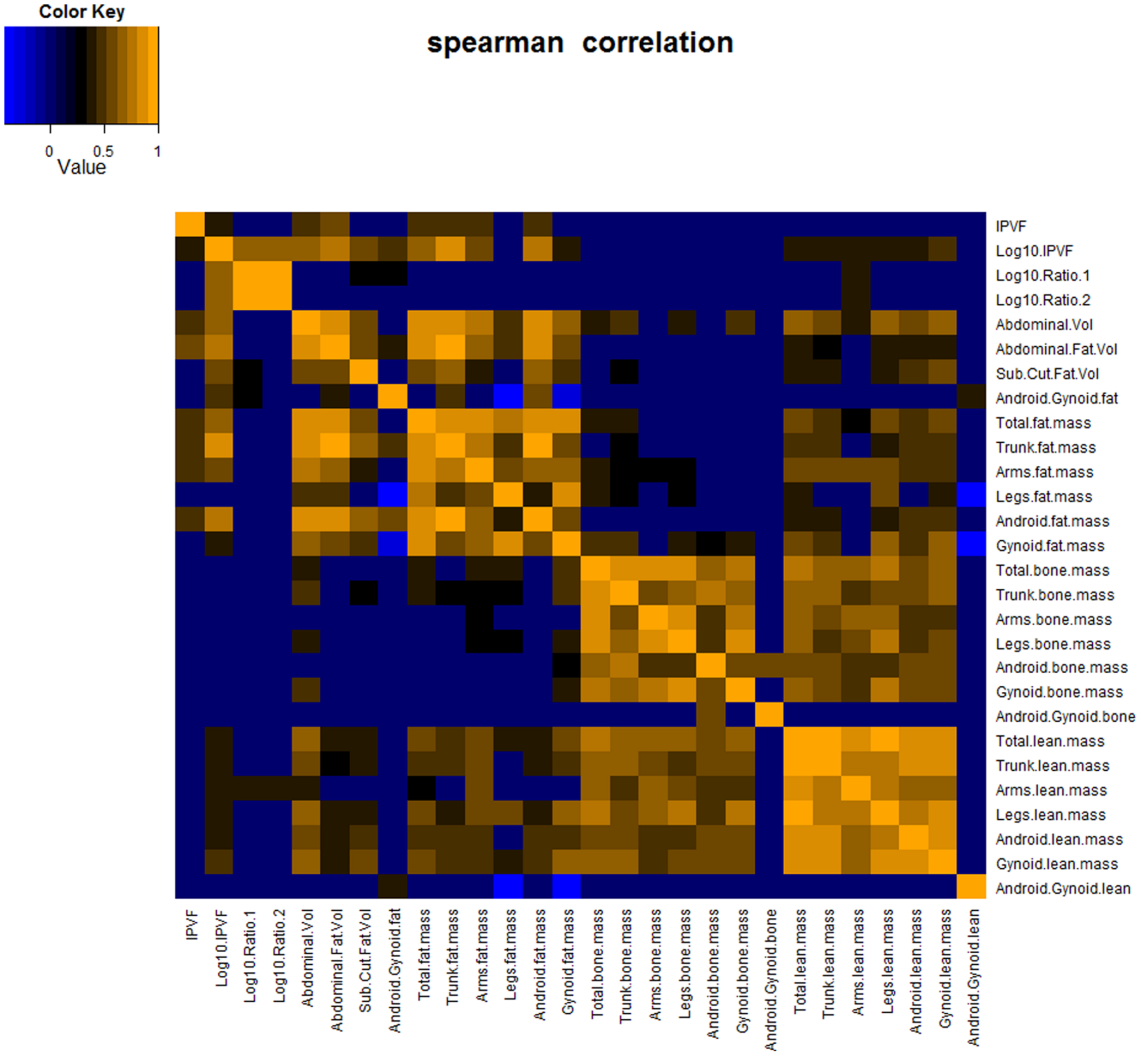
Statistically significant Spearman correlation map between CT scan and DXA body composition parameters (95% confidence interval). IPVF and Log_10_ IPVF correlated similarly to fat body composition parameters. Log_10_ IPVF showed a strong association with android/gynoid fat ratio (r = 0.48, p = 0.0015), Log_10_ VAT/SAT (r = 0.72, p<0.001) and Log_10_ VAT/total abdominal fat (r = 0.71, p<0.001), subcutaneous fat (r = 0.58, p<0.001), and some dependencies with lean mass parameters. Log_10_ values of VAT/SAT and VAT/total abdominal fat ratios were poorly correlated with most body composition parameters, except for arms lean mass (r = 0.43, p = 0.0056; r = 0.42, p = 0.0070), whilst VAT/SAT correlated with subcutaneous fat (r = 0.58, p = 0.0489), and android/gynoid fat ratio (r = 0.48, p = 0.0453). NB: Blue denotes negative correlation, orange denotes positive correlation, and black denotes no correlation.

PCA and a heat map based on the Spearman correlations on CT scan and DXA data were used to assess the relationships across the CT and DEXA generated body composition parameters (Figure 1, Figure S1). In particular, relationships with the different indicators of visceral adiposity including IPVF, log_10_ values of IPVF, ratio 1 and ratio 2, android/gynoid fat ratio were included. The analysis therefore described how visceral adiposity relates to region-specific lean, fat and bone distribution across the 40 human subjects according to the type of VAT indicator. Statistically significant correlations (95% confidence interval) are shown in Figure 1. The specific relationships between Log_10_ ratio 1 and the other parameters were further assessed using a multivariate OPLS model (Figure S2).

### Relationships between Visceral Fat Indicators and Clinical Parameters

**Figure 2:**
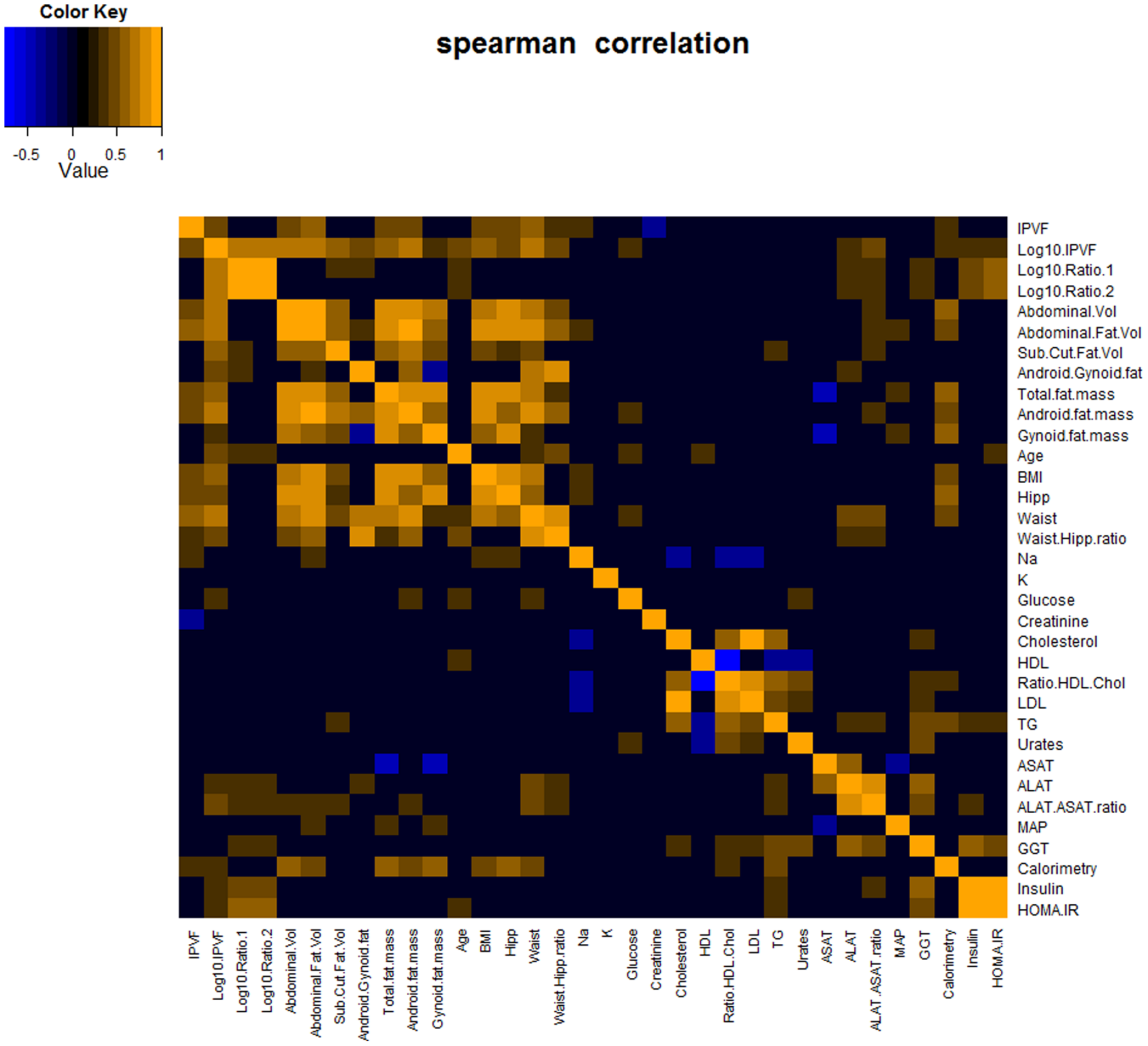
Statistically significant Spearman correlation map between body fat composition parameters and clinical measures (95% confidence interval). Log_10_ values of IPVF, VAT/SAT, VAT/total abdominal fat were strongly associated with HOMA-IR (r = 0.39, p = 0.015; r = 0.56, p<0.001; r = 0.55, p<0.001) and fasting insulin (r = 0.35, p = 0.0275; r = 0.49, p = 0.0017; r = 0.48, p = 0.0020). Strong associations were observed with ALAT (r = 0.39, p = 0.0128; r = 0.37, p = 0.0175; r = 0.38, p = 0.0167) and ALAT/ASAT ratio (r = 0.44, p = 0.0044; r = 0.35, p = 0.0268; r = 0.35, p = 0.0302). IPVF and Log_10_ values of IPVF correlated with waist (r = 0.55, p<0.001; r = 0.35, p = 0.04) and waist/hip ratio (r = 0.69, p<0.001; r = 0.52, p = 0.0017), but not Log_10_ values of VAT/SAT and VAT/total abdominal fat. NB: Blue denotes negative correlation, orange denotes positive correlation, and black denotes no correlation.

PCA and a heat map based on the Spearman correlations between body composition and main clinical parameters of the 40 human subjects were also employed to probe for relationships with key physiological endpoints (Figure 2, Figure S3). The specific relationships between Log_10_ ratio 1 and the other parameters were further assessed using a OPLS multivariate model (Figure S4).

### Visceral Adiposity Links to Different Metabolic Response to Oral Glucose Tolerance Test

Overall glucose and insulin response to OGTT correlated significantly with visceral adiposity as observed by linear regression with OGTT glucose and insulin AUC (Figure S5). When comparing subjects stratified according to VAT content defined quartiles, glucose-induced insulin secretion gradually increased between Q1 and Q4. However, significant blood glucose variations were only observed within the first 60 minutes post-glucose absorption across the VAT quartiles (Table 2, similar response was observed for different Log_10_ ratios).

**Table 2:** Insulin and glucose response to oral glucose tolerance test according to intraperitoneal/abdominal fat ratio.

Time variations	Delta t30–t0	Delta t60–t0	Delta t90–t0	Delta t120–t0
**Insulin concentrations (mU/mL)**				
** Q1**	74.69±31.76	85.94±49.76	75.25±41.38	38.93±32.32
** Q2**	77.93±33.43	97.04±41.48	62.87±34.48	54.27±31.97
** Q3**	79.25±52.36	68.07±42.78	91.01±55.36	66.61±32.39
** Q4**	108.80±49.86	114.764±64.45	108.73±49.17	70.92±29.56
** t-test p values (Q1 vs. Q4)**	0.085	0.278	0.117	0.033
Glucose concentrations (mmol/L)				
** Q1**	1.63±0.71	1.30±1.01	0.74±0.0.86	0.01±1.86
** Q2**	2.48±0.60	2.29±1.39	1.68±3.47	1.88±3.72
** Q3**	3.03±1.42	2.81±1.59	1.79±1.60	1.16±1.28
** Q4**	3.21±0.93	3.33±1.6	1.95±2.00	1.09±2.05
** t-test p values (Q1 vs. Q4)**	0.0005	0.0006	0.096	0.2355
Estimates of beta cell function and insulin sensitivity OGTT Delta(Ins t30–t0)/(Gly t30–t0)				
** Q1**	52.09±26.56			
** Q2**	31.16±12.20			
** Q3**	39.42±32.53			
** Q4**	41.81±40.61			
** t-test p values (Q1 vs. Q4)**	0.511			

NB: Values are reported as mean values ± SD. Key: Qi: data for population quartile i according to intraperitoneal/abdominal fat ratio.

### Plasma Metabolic Profiles Revealed a Lipid and Amino Acid Signature Associated with Visceral Adiposity

In order to identify phenotypic signatures of visceral fat deposition, plasma samples were analysed using ^1^H-NMR and targeted LC-MS/MS metabonomic approach. Analyses were conducted on the fasting plasma samples collected at V0 and V2. OPLS analysis of ^1^H-NMR samples collected at V0 and V2 showed some subtle but significant associations between blood plasma lipids and visceral fat deposition (R^2^X: 0.68; R^2^Y: 0.506; Q^2^Y: 0.167), further confirmed by random forest analysis (Figure S6). These results suggested plasma lipid remodelling marked by changes in glycerophospholipids and fatty acid saturation patterns.

**Figure 3:**
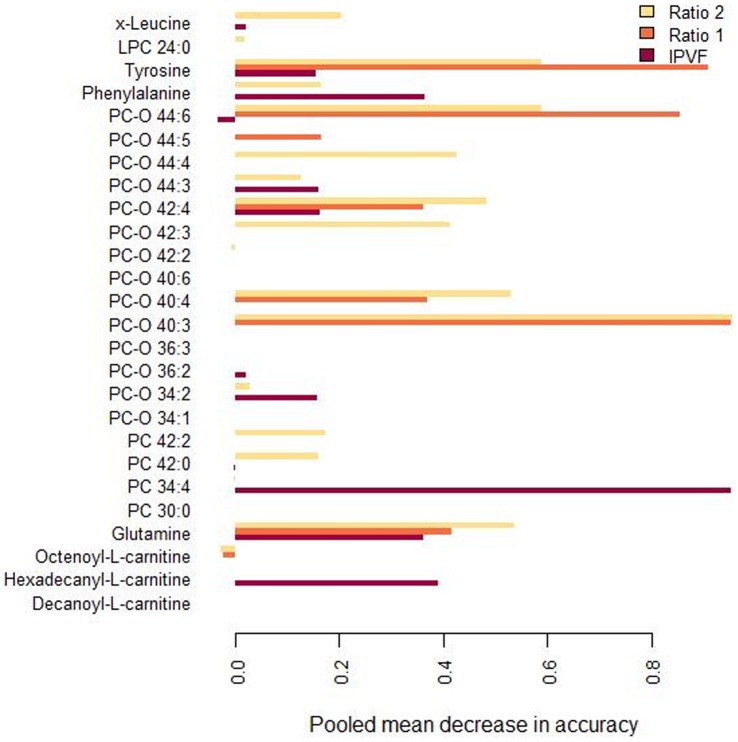
Plot describing metabolite importance and robustness in predicting visceral fat adiposity as assessed by Random forest analysis using metabolic data collected at V0 and V2. Visceral adiposity was associated with increasing concentrations of amino acids (glutamine, leucine/isoleucine, phenylalanine and tyrosine), lysophosphatidylcholine LPC 24∶0 and diacyl phospholipids (PC 30∶0, PC 34∶4). In addition, visceral adiposity was marked by a depletion in ether lipid species PC*-O* 36∶3, PC*-O* 40∶3, PC*-O* 40∶4, PC*-O* 40∶6, PC*-O* 42∶2, PC*-O* 42∶3, PC*-O* 42∶4, PC*-O* 44∶3, PC*-O* 44∶4, PC*-O* 44∶6, and two diacyl phosphocholines (PC 42∶0 and PC 42∶2). To reflect the weight of the selected biomarkers in the classification of visceral adiposity, a pooled mean decrease of accuracy for each compound was calculated from 10000 forest generations. Higher variable importance corresponds to higher values of pooled mean decrease in accuracy. Key: IPVF, intraperitoneal fat volume; LPC, Lysophosphatidylcholines; PC, Phosphatidylcholines; PC-O, 1-O-alkyl-2- acylglycerophosphocholines; Ratio1, intraperitoneal/subcutaneous fat ratio; Ratio 2, intraperitoneal/abdominal fat ratio. Assignment of PC-O species is made on the assumption that only even numbered carbon chains are present. A potential overlap between PC species containing odd-chain fatty acids and even-chained PC-O species cannot be excluded with low mass resolution.

Targeted MS metabonomics provided quantitative measures of 163 metabolites, including amino acids, sugars, acyl-carnitines, sphingolipids, and glycerophospholipids in plasma samples. A metabolic signature of visceral fat adiposity was determined using an OPLS analysis across the 40 individual subjects (R^2^X: 0.29; R^2^Y: 0.68; Q^2^Y: 0.32). The model was then tested for assessing inter-days metabolic variations, by considering each visit separately and by modelling potential inter-days metabolic differences (data not shown). No statistically significant differences were observed in the blood plasma metabolic profile between V0 and V2 (data not shown). Furthermore, the most robust metabolic signatures of visceral fat status using either value of the intraperitoneal fat volume, ratio 1 and ratio 2, were selected by Random Forests analysis. Using this methodology, metabolite importance and robustness in predicting visceral fat adiposity were assessed using metabolic data collected at V0 and V2 (Figure 3). A signature of specific amino acids, diacyl phospholipids and ether lipid species was identified and shown to be preserved significantly between days (Figure 3). Ultimately, 26 metabolites were retained (Figure 3, Table 3, Table S3 and Table S4). Metabolite importance and robustness in predicting visceral fat adiposity as assessed by Random forest analysis using metabolic data collected at V0 and V2, highlighted 7 most robust markers, including tyrosine, glutamine, PC-*O* 44∶6, PC-*O* 44∶4, PC-*O* 42∶4, PC-*O* 40∶4, and PC-*O* 40∶3.

**Table 3:** Metabolite variations across subjects stratified according to intraperitoneal/abdominal fat ratio.

Metabolites (concentration)	Q1	Q2	Q3	Q4	Mann-Whitney p value (Q1/Q4)
PC-O 42∶4*, µmol/L	1.34±0.33	1.09±0.28	1.09±0.37	0.82±0.22	0.00298
PC-O 40∶3*, µmol/L	1.41±0.27	1.46±0.38	1.27±0.3	0.86±0.42	0.00421
PC-O 44∶6*, µmol/L	1.52±0.56	1.22±0.3	1.11±0.5	1.03±0.32	0.01013
PC-O 44∶4*, µmol/L	0.8±0.3	0.67±0.24	0.63±0.19	0.51±0.18	0.01721
PC-O 40∶4*, µmol/L	2.79±0.56	2.9±0.73	2.47±0.69	2.02±0.83	0.01784
Glutamine, µmol/L	615.56±107.95	748±193.49	792.1±260.61	714±94.03	0.02468
PC-O 36∶3*, µmol/L	7.04±1.68	6.7±2.61	7.11±1.82	5.5±1.24	0.02792
8-iso-PGF2α, ng/100 µL	0.008±0.011	0.004±0.001	0.003±0.002	0.003±0.001	0.0370
Phenylalanine, µmol/L	49.9±14.16	50.47±8.45	62.82±22.17	56.42±8.38	0.04113
PC-O 44∶5*, µmol/L	2.29±0.74	2.03±0.55	1.9±0.74	1.71±0.7	0.04113
Leucine+Isoleucine, µmol/L	181.44±53.02	214.2±56.71	202.4±27.39	228.8±33.83	0.04536
Tyrosine, µmol/L	61.97±11.02	80.54±22.21	75.91±21.83	80.99±24.69	0.05347
PC-O 42∶2*, µmol/L	0.66±0.23	0.56±0.14	0.53±0.16	0.45±0.18	0.05347
PC-O 40∶6*, µmol/L	3.81±0.86	3.27±1.09	2.8±0.84	2.74±1.09	0.06525
PC-O 42∶3*, µmol/L	0.89±0.2	0.92±0.14	0.9±0.27	0.63±0.26	0.06525
PC-O 36∶2*, µmol/L	11.29±2.64	11.86±2.68	10.38±3.08	9.17±2.09	0.07865
PC 42∶0, µmol/L	0.65±0.23	0.48±0.16	0.47±0.08	0.48±0.14	0.07889
Palmitoylcarnitine, µmol/L	0.07±0.02	0.07±0.03	0.07±0.03	0.10±0.04	0.12065
PC 30∶0, µmol/L	4.43±1.48	5.17±2.35	5.75±1.98	5.57±1.76	0.1564
PC-O 44∶3*, µmol/L	0.21±0.06	0.2±0.04	0.15±0.06	0.17±0.05	0.1564
PC-O 34∶1*, µmol/L	9.94±2.22	9.78±3.84	8.48±2.15	8.53±0.99	0.17752
15-HETE, ng/100 µL	0.08±0.05	0.17±0.29	0.1±0.13	0.1±0.18	0.18231
PC-O 34∶2*, µmol/L	10.66±3.5	9.31±3.51	9.38±4.57	8.77±1.76	0.21102
LPC 24∶0, µmol/L	0.36±0.25	0.51±0.24	0.52±0.36	0.46±0.31	0.21613
Octenoylcarnitine, µmol/L	0.04±0.02	0.05±0.02	0.05±0.04	0.05±0.02	0.25258
9-HODE, ng/100 µL	0.10±0.02	0.12±0.05	0.11±0.02	0.12±0.04	0.25831
PC 34∶4, µmol/L	1.3±0.46	1.53±1.14	1.41±0.52	1.55±0.75	0.31537
Caproylcarnitine, µmol/L	0.22±0.10	0.2±0.09	0.14±0.06	0.30±0.19	0.40018
PC 42∶2, µmol/L	0.2±0.06	0.19±0.11	0.13±0.05	0.17±0.08	0.40018
12-HETE, ng/100 µL	0.60±0.72	0.28±0.3	1.15±2.15	0.41±0.36	0.8421
AA, ng/100 µL	784.22±236.64	764.9±314.46	854.7±193.24	785.6±160.86	0.96823

NB: Blood plasma metabolites highlighted by multivariate analyses are reported as mean values ± SD. Key: Qi: data for population quartile i according to intraperitoneal/abdominal fat ratio. 12-HETE, 12-hydroxy-eicosatetraenoic acid; 15-HETE, 12-hydroxy-eicosatetraenoic acid; 9-HODE, 9-Hydroxy-10,12-octadecadienoic acid; AA, arachidonic acid; LPC, Lysophosphatidylcholines; PC, Phosphatidylcholines; PC-O, 1-O-alkyl-2- acylglycerophosphocholines; SM, Sphingomyelines; SM-OH, Hydroxy-Sphingomyelin.

* Assignment of PC-O species is made on the assumption that only even numbered carbon chains are present. A potential overlap between PC species containing odd-chain fatty acids and even-chained PC-O species cannot be excluded with low mass resolution.

**Figure 4:**
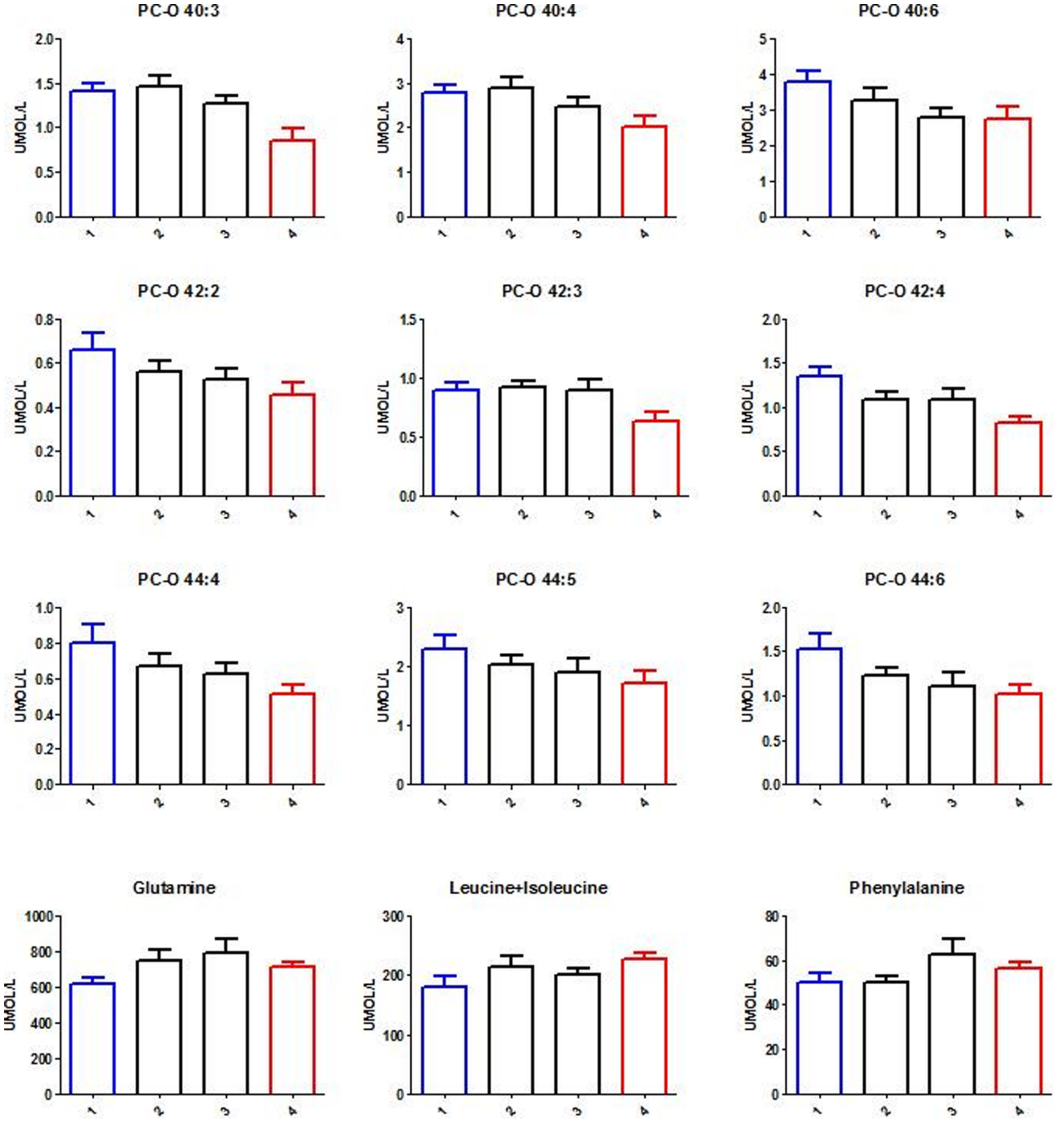
Bar plots describing metabolite variations in the study population stratified in four quartiles according to visceral fat adiposity (intraperitoneal fat) at V2. Statistical significance is reported in Table S3. Key: PC-O, 1-O-alkyl-2- acylglycerophosphocholines. Assignment of PC-O species is made on the assumption that only even numbered carbon chains are present. A potential overlap between PC species containing odd-chain fatty acids and even-chained PC-O species cannot be excluded with low mass resolution.

To assess potential overlaps with insulin resistance, the predictive performance of the seven most promising markers was evaluated using two linear discriminant classification models based on both visceral fat and HOMA-IR stratification of the population. Model on visceral fat showed a sensitivity and specificity in cross-validation of 0.90, which was fairly different from the model on HOMA-IR (sensitivity (CV): 0.95, specificity (CV): 0.68). This loss of specificity for the HOMA-IR model was interpreted as a stronger association of the selected markers with visceral adiposity. Figure 4 displays metabolite variations in the study population stratified in four quartiles according to visceral fat adiposity (Log_10_ value of intraperitoneal fat).

### Eicosanoid Profiling Suggests that the Relationship between Visceral Adiposity, Inflammation and Oxidative Stress is Affected by other Environmental Confounders

To investigate relationships between visceral fat deposition and low-grade inflammatory status, a targeted LC-MS/MS method was employed to measure plasma eicosanoid concentrations at V0 and V2. Here, Random Forests analysis on quantitative data displayed statistical relevant changes associated with the visceral adiposity (Table 3, Figure S7, Table S3 and Table S4).

### Urine Metabolic Profiles Suggested a Different Energy and Microbial Metabolism in Subjects with High Visceral Adiposity

^1^H-NMR spectra of the 24 hours urine samples were normalized to creatinine concentration and were analyzed using supervised chemometric analysis to assess the occurrence of relationships with visceral adiposity. PLS analyses using 2 predictive components showed relationships between urine metabolic profile and visceral adiposity, at both timepoints, V1 or V2 (respective Q^2^Y values being 0.05, 0.217 and 0.54). OPLS analysis confirmed a statistically significant relationship at V2 (1 predictive and 2 orthogonal components, Q^2^Y value of 0.27). Visceral adiposity was correlated with changes in central energy metabolism (increased urinary excretion of 3-methyl-2-oxovaleric acid (direct catabolic product of isoleucine), N1-methyl-2-pyridone-5-carboxamide (2PY), N1-methyl-4-pyridone-3-carboxamide (4PY), fucose, pyruvate, ethanolamine and lactate), protein metabolism (increased pseudouridine, decreased taurine, and 3-methyl-histidine), and gut microbial activities (decreased 4-cresol sulfate). Since the subjects received a controlled dietary intake during V2, these results suggest a strong incident of visceral fat adiposity on individual nutritional response as noted here.

### Integration of Visceral Adiposity, Clinical Parameters and Metabonomics Markers

**Figure 5:**
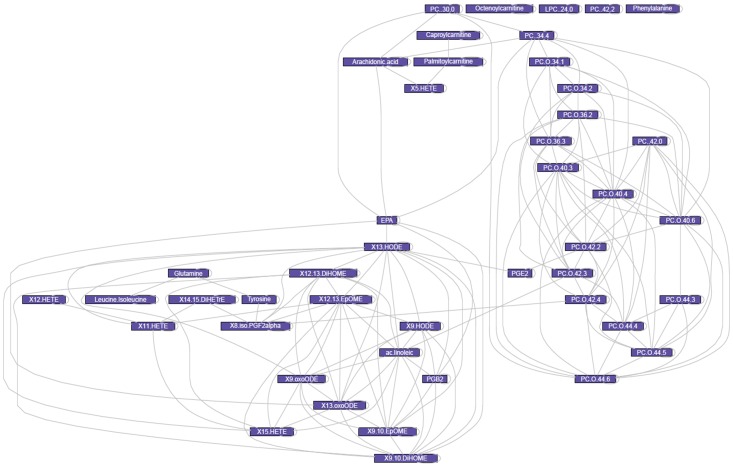
Spearman correlation network between blood plasma metabolic markers highlighting strong functional relationships between phospholipids and eicosanoid metabolic remodelling. Non significant correlations and those between 0.4 and −0.4 were removed to reduce the number of edges and facilitate visualization.

A heat map based on the Spearman correlations on CT scan, main clinical parameters and selected discriminant metabolites measured in plasma and urine of the 40 human subjects was used to assess the relationships between the urine and plasma metabonomics readouts and key physiological endpoints. These correlations are shown in Figure 5 and Figure S8. Such an approach enabled the assessment of linear relationships between the parameters across the 40 subjects. In addition, for the 26 most influential metabolites, the VIP values generated from PLS regression against the visceral fat estimates are reported in Table S5, to further exemplify these relationships. In particular, we investigated the statistical relationships between selected metabolites, HOMA-IR, ALAT/ASAT ratio and visceral adiposity (Figure 5). The spearman correlation network between blood plasma metabolites showed a strong connection between the remodelling of ether lipid species and specific diacyl phospholipids, which seem quite independent of changes in eicosanoids and amino acids.

## Discussion

Over the last decades, there was increasing awareness that prevalence of fat storage in the trunk/android compartment over gynoid (i.e. mainly subcutaneous) region associates with increased insulin resistance and related cardiometabolic risk [36], [37]. Assessments of visceral adiposity using VAT/SAT (ratio 1) and VAT/total abdominal fat (ratio 2) ratios were conducted in the present study, as they are known to better correlate to cardiometabolic health, outperforming associative power of single BMI and VAT [29]. In our cohort, IPVF, Log_10_ IPVF and waist/hip ratio correlated similarly to several fat body composition parameters (total, trunk, arms, android and abdominal fat), concurring with literature [26], [36], [37]. VAT/SAT and VAT/total abdominal fat ratios were independent of these variables, and also related to android/gynoid fat ratio. Visceral adiposity expressed by log_10_ values of IPVF, ratios 1 and 2 associated significantly with insulin (fasting insulin, HOMA-IR) and hepatic (ALAT/ASAT) metabolism unlike with other clinical parameters, including HDL, LDL, and TG, which is consistent with the healthy status of the enrolled subjects.

### Ether Lipid Metabolic Signature of Visceral Adiposity Highlights Potential Deregulations in Lipoprotein and Phospholipid Metabolic Pathways

A visceral fat signature of specific amino acids and ether phosphocholine lipid species was shown to be preserved significantly between days (Figure 3). Ether lipid species represent 18% of the total pool of phospholipids and are mainly composed by plasmalogens. Although little is known about their systemic metabolism, plasmalogens have been implicated in protection of cellular functions against oxidative damage [38], and their diminished levels have been reported in several diseases [39], *e.g.* diabetes mellitus, vascular diseases and obesity [40] or peroxisome biogenesis defects [41], all these conditions exhibiting a common feature as regards with oxidative stress. Furthermore, our work supports the association of polyunsaturated phospholipids species with visceral adiposity [40]. Using a correlation analysis, PC-*O* species reduced with increased visceral fat adiposity and positively correlated to HDL circulating levels, a feature previously reported for ageing-associated cardiometabolic disorders [42] and lipoprotein associated with visceral adiposity [26], [43], [44]. Indeed, lipoprotein fractions (VLDL, LDL, and HDL) were previously characterized by a specific ether lipid class and species pattern [39]. Our results confirm the involvement of PC-*O* species in glucose metabolism providing an additional pattern of PC-*O* species (PC-*O* 40∶3, PC-*O* 40∶4, PC-*O* 42∶4, PC-*O* 44∶3, and PC-*O* 44∶6) newly associated with visceral adiposity. Further correlation analysis showed significant association between HOMA-IR and circulating levels of PC-*O* 44∶4, PC-*O* 44∶5, PC-*O* 44∶6 and PC-*O* 42∶4. Floegel et al. reported a pattern of PC-*O* species (PC-*O* 34∶3, PC-*O* 40∶6, PC-*O* 42∶5, PC-*O* 44∶4, and PC-*O* 44∶5) correlated with risk of T2D [45]. Taken together with literature data, our results suggest the occurrence of a subset of PC-*O* species (PC-*O* 40∶3, PC-*O* 40∶4, and PC-*O* 44∶3) that would be specific to visceral adiposity. Additionally, we observe that PC-*O* 42∶4, PC-*O* 44∶4, PC-O 44∶5 and PC-*O* 44∶6 appear as constant feature of insulin resistance status or Type 2 pre-diabetic state.

Our results further supported by the recent study by Szymanska et al. [26], strongly suggest a remodelling of phospholipid species, which may result from a multi-factorial origin, including dietary factors, gut functional ecology, intestinal absorption, as well as Platelet-Activating Factor (PAF) metabolic pathways and phospholipase A2 activities [46], [47], which are modulated by obesity and insulin resistance [3], [48].

Evidence tends to support the notion that oxidative-stress-induced dysregulation of inflammation and adipokines may mediate the obesity-related metabolic derangement [49]. Here, high visceral fat subjects tend to display a different balanced network of lipid mediators, as noted by an alteration of the arachidonic metabolism. However, the greater inter-day variability in the dynamics of these pathways may suggest that the visceral-associated influence is also dependent on lifestyle and dietary intakes. Previously reported studies displayed how enhanced lipid peroxidation and persistent platelet activation associate to visceral adiposity might be influenced by weight-loss program or simply by the type of fat in meals [3], [50]. Here, a decreased circulating level of 8-iso-PGF2α was highlighted, a feature negatively correlated with the visceral fat ratio and android fat across the 40 subjects and positively with blood pressure. Others have characterized how increased blood and urinary levels of 8-iso-PGF2α correlate with oxidative stress and android obesity [3], [51]. 8-iso-PGF2α is generally released from the site of inflammation as esters of phospholipid (bound) or through the action of phospholipase A2 in free form [52]. 8-iso-PGF2α concentration was positively correlated with the concentrations of several ether lipids decreasing with visceral adiposity (e.g. PC-O 40∶4, PC-O 42∶2, PC-O 42∶3, PC-O 42∶4). Furthermore, whilst 12- and 15-LO enzymes are known to be up-regulated in visceral adipocytes, their products (12- and 15- hydroxy-eicosatetraenoic acid 12/15-HETE) promote proinflammatory state and impairing insulin signaling in adipocytes [4], [53]. However, the circulating levels of 12/15-HETE concentrations were not statistically different between groups, suggesting that the circulating levels of these molecules in fasting blood are not directly related to visceral fat metabolism under the current experimental conditions.

### Visceral Adiposity Links to a Specific Amino Acid Pattern Associated with Deregulated Insulin Signaling

Plasma amino acids (including glutamine, leucine/isoleucine, phenylalanine and tyrosine) significantly contributed to the metabolic phenotypes of visceral adiposity, with a greater specificity being observed for glutamine and tyrosine levels. Our findings are partly supported by a recent report on obese Japanese subjects in which plasma levels of alanine, glycine, glutamate, tryptophan, tyrosine and BCAA amino acids associated with visceral fat accumulation [27]. Our results support associations of levels of BCAA and tyrosine with visceral adiposity, irrespective of ethnicity, lifestyle and environmental conditions. Although requiring proper validation on a larger population, one may postulate that levels of glycine, glutamate, tryptophan, glutamine and phenylalanine associated with visceral adiposity could be genetic and environmental dependent either in healthy obese [27] or in pre-diabetic subjects as recently reported [54]. Association of BCAA levels with visceral adiposity is supported by previous reports proposing these as co-variants of insulin resistance [55], [56]. In fact, BCAA catabolism is tightly intertwined with insulin resistance, and greater circulating levels of BCAA were reported under these conditions [57], [58]. In particular, a combination of three amino acids (isoleucine, phenylalanine, tyrosine) could predict future diabetes (>5-fold higher risk for individuals in top quartile) [59], [60]. Recent results showed that fat tissue metabolism is key in determining the blood level of branch chain amino acids [61]. Indeed, it is suggested that altered signaling in white adipose tissue under insulin resistance, pre-diabetes or type 2 diabetes conditions can induce decreased expression of branched-chain-keto acid dehydrogenase (BCKD) with inferred impairment of BCAA utilization as metabolic fuel in this tissue. As a result, one may postulate that this reduced BCAA utilization in adipose tissue increases the circulating pool of isoleucine and its direct catabolic product, the 3-methyl-2-oxovaleric acid, via reversible transamination by branched chain amino-acid transaminase 1. This may explain the observed increased urinary excretion of 3-methyl-2-oxovaleric acid in high visceral fat subjects.

In the present study, visceral adiposity was associated with different response to OGTT, as noted with an early increase in post-glucose insulin concentrations and enhanced glucose-induced insulin secretion. Knowing that insulin resistance is a causal mechanism in the etiology of visceral fat development and related disorders, the observation of such amino acid and OGTT patterns support this functional relationship. Moreover, removal of intraperitoneal fat tissue, a major compartment of visceral adiposity, significantly restores glucose and insulin towards normal levels in humans, but not subcutaneous adipose tissue [22].

The study presents several potentialities, including the use of CT and DXA measurements to provide robust biological readouts to compare the association between different estimates of visceral adiposity. Then, the subjects were recruited from a well-defined population, which represented a single ethnic group under well-defined conditions and healthy medical criteria. This study also has several limitations, due to exploratory nature and the use of a single cohort study with a small number of subjects and the results are confined to this specific cohort. Therefore, further studies are needed to determine the predictive role of the highlighted metabolic signature clustering of cardiometabolic risk factors and subsequent incidence of cardiovascular diseases. Since the metabolic signatures encompass features related to glucose/insulin metabolism, future studies should investigate closely the relationships between metabolism, visceral adiposity and glucose tolerance classes. Moreover, there is compelling evidence that relationships between abdominal fat distribution and insulin and non-insulin-mediated glucose uptake in females are dependent on endogenous androgens [62], whilst the global adiposity and thickness of intraperitoneal and mesenteric adipose tissue depots have been found increased in women with polycystic ovary syndrome [63]. It will be therefore key in future studies to comprehensively integrate endocrinopathy with metabonomics and other physiological parameters.

## Conclusions

The integration of visceral fat estimates with metabolic profiles of blood and urine described a distinct amino acid, diacyl and ether phospholipid phenotype associated with higher visceral fat in a healthy obese women cohort. Metabolite importance and robustness in predicting visceral fat adiposity as assessed by Random forest analysis highlighted 7 most robust markers, including tyrosine, glutamine, PC-*O* 44∶6, PC-*O* 44∶4, PC-*O* 42∶4, PC-*O* 40∶4, and PC-*O* 40∶3.Moreover, the inflammatory intermediate profile appears of lower relevance for clinical assessment of visceral fat due to greater inter-days and between-subject variations. Considering the recent studies regarding at metabolic processes associated with different body fat distributions, it becomes imperative that the following studies comprehensively report anthropometric, endocrine, clinical and medical details of the cohort, in addition to race/ethnic and age variability. This will allow a stronger link to unravel metabolic susceptibilities to metabolic syndrome and cardiovascular health. Such reports will enable proper comparison of metabolic findings and follow-up in larger populations. In future, systemic metabonomic visceral adiposity biomarkers might also be extended to distinguish its mesenteric, epicardial and peripheric deposition subtypes which may link even more specifically to cardiometabolic status of patients with disorders or at risk of diseases.

## Supporting Information

Figure S1 **Loadings plot from Principal component analysis of CT and DEXA body composition parameters.** First two pprincipal components explained 42 and 17% of the total variance.
(TIF)

Figure S2 **Loadings plot from orthogonal partial least square analysis of Log10 value of ratio 1 with CT and DEXA body composition parameters.** OPLS model was generated with 1 predictive and 2 orthogonal components (R^2^X = 0.60, R^2^Y = 0.96, Q^2^Y = 0.90).
(TIF)

Figure S3 **Loadings plot from Principal component analysis of CT, DEXA body composition and clinical parameters.** First two pprincipal components explained 29 and 13% of the total variance.
(TIF)

Figure S4 **Loadings plot from orthogonal partial least square analysis of Log10 value of ratio 1 with CT and DEXA body composition parameters.** OPLS model was generated with 1 predictive and 2 orthogonal components (R^2^X = 0.45, R^2^Y = 0.92, Q^2^Y = 0.75).
(TIF)

Figure S5 **Linear regression between glucose response to OGTT and visceral adiposity.**
(TIF)

Figure S6 **Statistical reconstruction of ^1^H NMR blood plasma profiles using random forest analysis to identify metabolic patterns associated with visceral adiposity (as identified with squared boxes).** GPCs =  glycerophospholipids, PUFAs =  polyunsaturated fatty acids, UFAs =  unsaturated fatty acids.
(TIF)

Figure S7 **Scheme summarizing metabolic differences in the arachidonic and linoleic acid metabolic pathway across the four groups of visceral adiposity (Q1, Q2, Q3, and Q4).** Bar plots describing metabolite variations in the study population stratified in four quartiles according to visceral fat adiposity (intraperitoneal fat). Statistical significance is reported in Table S2. Key: Qi: data for population quartile i according to intraperitoneal/abdominal fat ratio. 12-HETE, 12-hydroxy-eicosatetraenoic acid; 15-HETE, 12-hydroxy-eicosatetraenoic acid; 9-HODE, 9-Hydroxy-10,12-octadecadienoic acid; AA, arachidonic acid.
(TIF)

Figure S8 **Statistically significant Spearman correlation map between visceral fat parameters CT scan, DXA data and main clinical parameters (95% confidence interval).** HOMA-IR is negatively correlated with PC*-O* 42∶4, PC*-O* 44∶4, and PC*-O* 44∶6 lipid species, and positively with blood triglycerides, γGT, and age. ALAT/ASAT ratio showed positive correlations with AA and EPA, PC 34∶4, PC 30∶0 and LPC 24∶0 lipid species, but also fasting blood insulin, γGT and triglycerides, waist circumference and waist to hip ratio. Blue denotes negative correlation, orange denotes positive correlation, and black denotes no correlation.
(TIF)

Table S1 **Descriptive statistics of subjects stratified according to intraperitoneal fat volume.**
(DOCX)

Table S2 **Descriptive statistics of subjects stratified according to intraperitoneal/subcutaneous fat ratio.**
(DOCX)

Table S3 **Metabolite variations across of subjects stratified according to intraperitoneal fat volume.**
(DOCX)

Table S4 **Metabolic variations across subjects stratified according to intraperitoneal/subcutaneous fat ratio.**
(DOCX)

Table S5 **Metabolite VIP ranking based on PLS models generated at V2+V0.**
(DOCX)

Text S1 **Sample Preparation and ^1^H-NMR Spectroscopic Analysis.**
(DOCX)

Text S2 **Sample preparation for Biocrates Life Sciences Absolute** ***IDQ*** **™ kit analysis.**
(DOCX)

Text S3 **Sample preparation and inflammation markers quantification by UPLC-ESI-MS/MS using isotope dilution technique.**
(DOCX)
