# Correction: Topographical Body Fat Distribution Links to Amino Acid and Lipid Metabolism in Healthy Obese Women

**DOI:** 10.1371/annotation/1c88ae20-07d1-4fda-8cd8-058d95af057d

**Published:** 2013-09-24

**Authors:** Francois-Pierre J. Martin, Ivan Montoliu, Sebastiano Collino, Max Scherer, Philippe Guy, Isabelle Tavazzi, Anita Thorimbert, Sofia Moco, Megan P. Rothney, David L. Ergun, Maurice Beaumont, Fiona Ginty, Salah D. Qanadli, Lucie Favre, Vittorio Giusti, Serge Rezzi

There was an error in the title of the article. The correct version of the title in the article is: Topographical Body Fat Distribution Links to Amino Acid and Lipid Metabolism in Healthy Obese Women

The correct citation is: Martin F-PJ, Montoliu I, Collino S, Scherer M, Guy P, et al. (2013) Topographical Body Fat Distribution Links to Amino Acid and Lipid Metabolism in Healthy Obese Women. PLoS ONE 8(9): e73445. doi:10.1371/journal.pone.0073445 

