# Supplementary figures and images for: Topographical Body Fat Distribution Links to Amino Acid and Lipid Metabolism in Healthy Non-Obese Women
Source: PLoS One. 2013 Sep 11;8(9):e73445. doi: 10.1371/journal.pone.0073445 (PMC3770640; doi:10.1371/journal.pone.0073445)

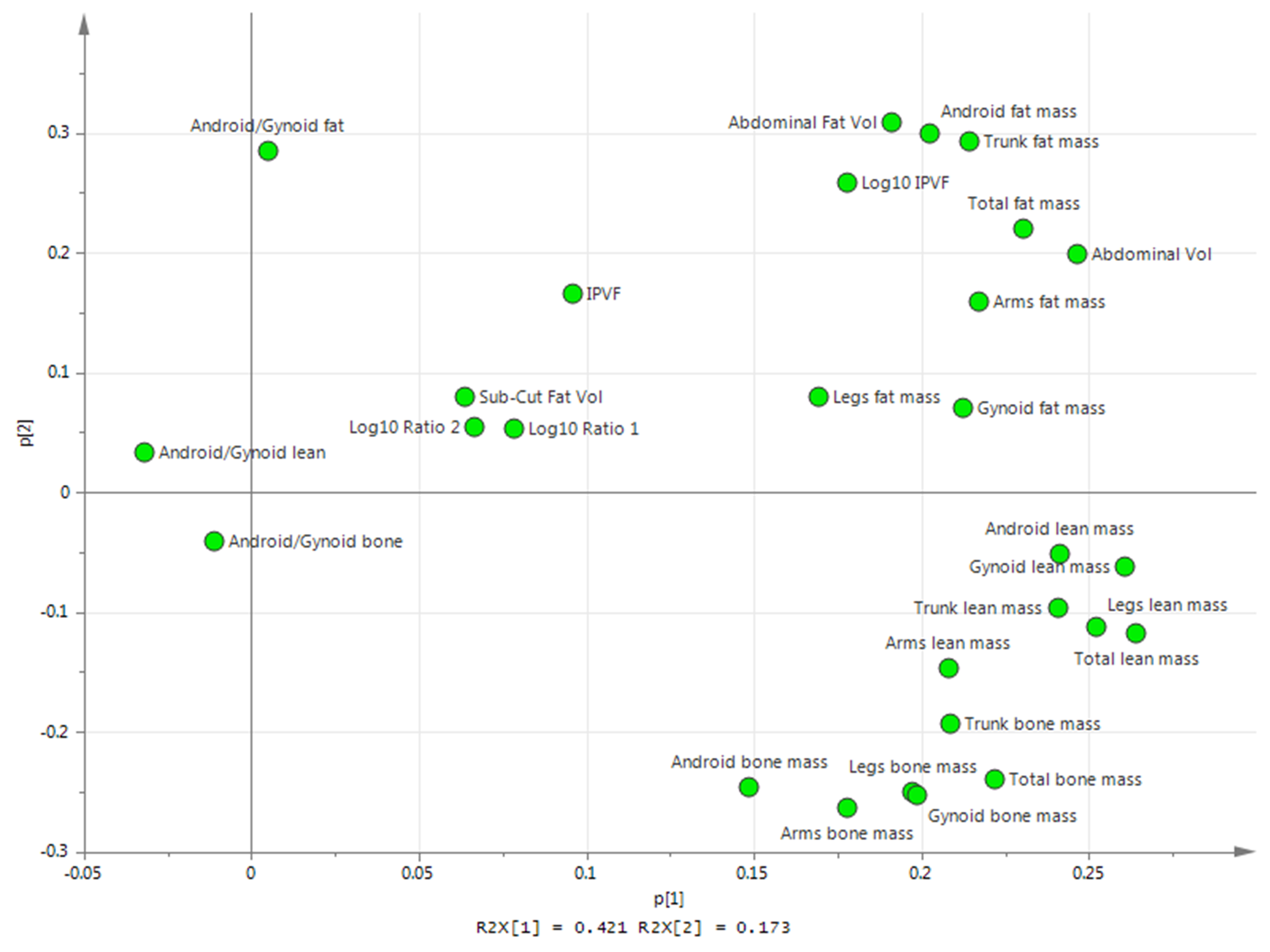

Supplement: Figure S1 — Loadings plot from Principal component analysis of CT and DEXA body composition parameters. First two pprincipal components explained 42 and 17% of the total variance. (TIF) [file pone.0073445.s001.tif]

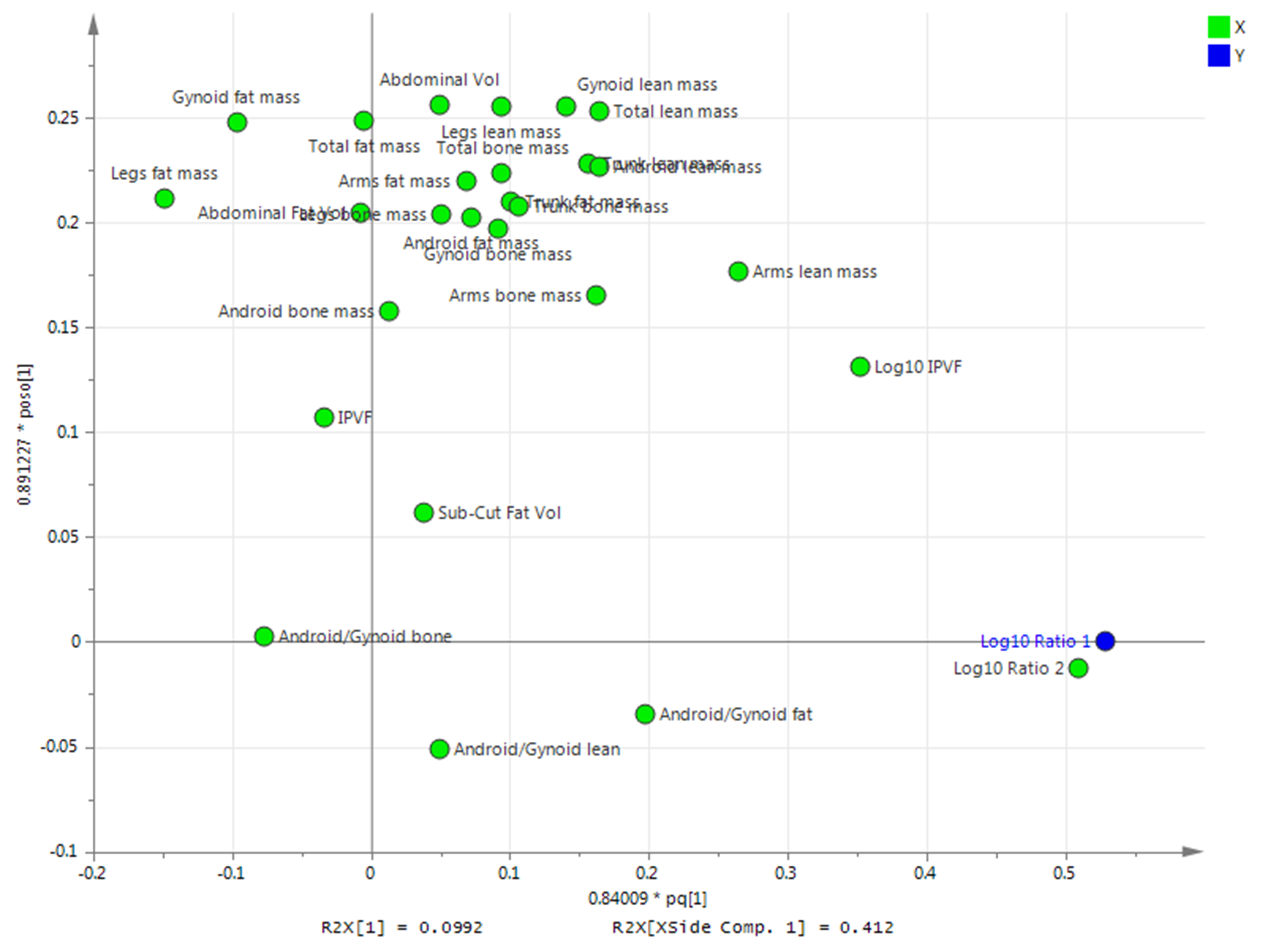

Supplement: Figure S2 — Loadings plot from orthogonal partial least square analysis of Log10 value of ratio 1 with CT and DEXA body composition parameters. OPLS model was generated with 1 predictive and 2 orthogonal components (R2X = 0.60, R2Y = 0.96, Q2Y = 0.90). (TIF) [file pone.0073445.s002.tif]

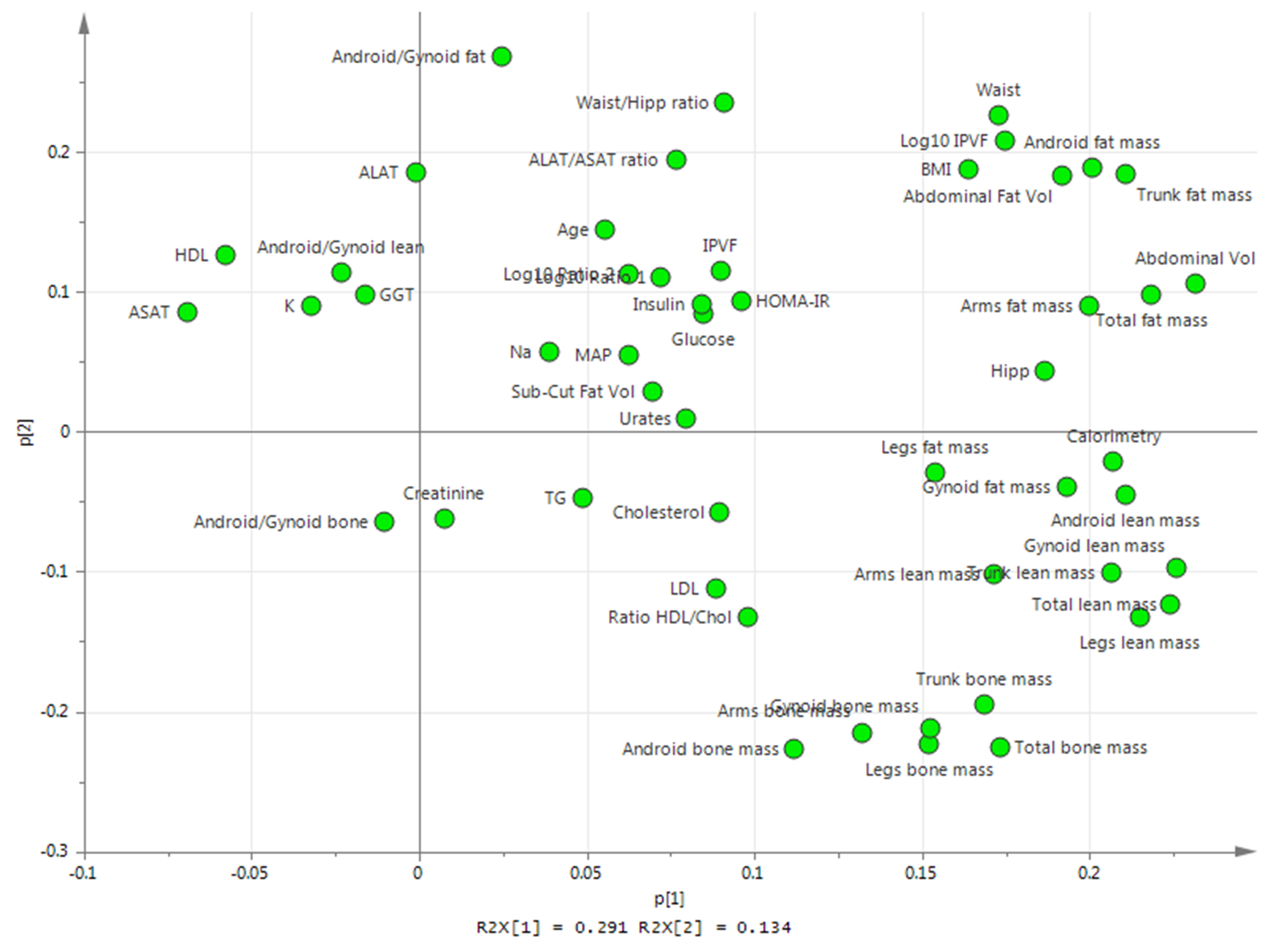

Supplement: Figure S3 — Loadings plot from Principal component analysis of CT, DEXA body composition and clinical parameters. First two pprincipal components explained 29 and 13% of the total variance. (TIF) [file pone.0073445.s003.tif]

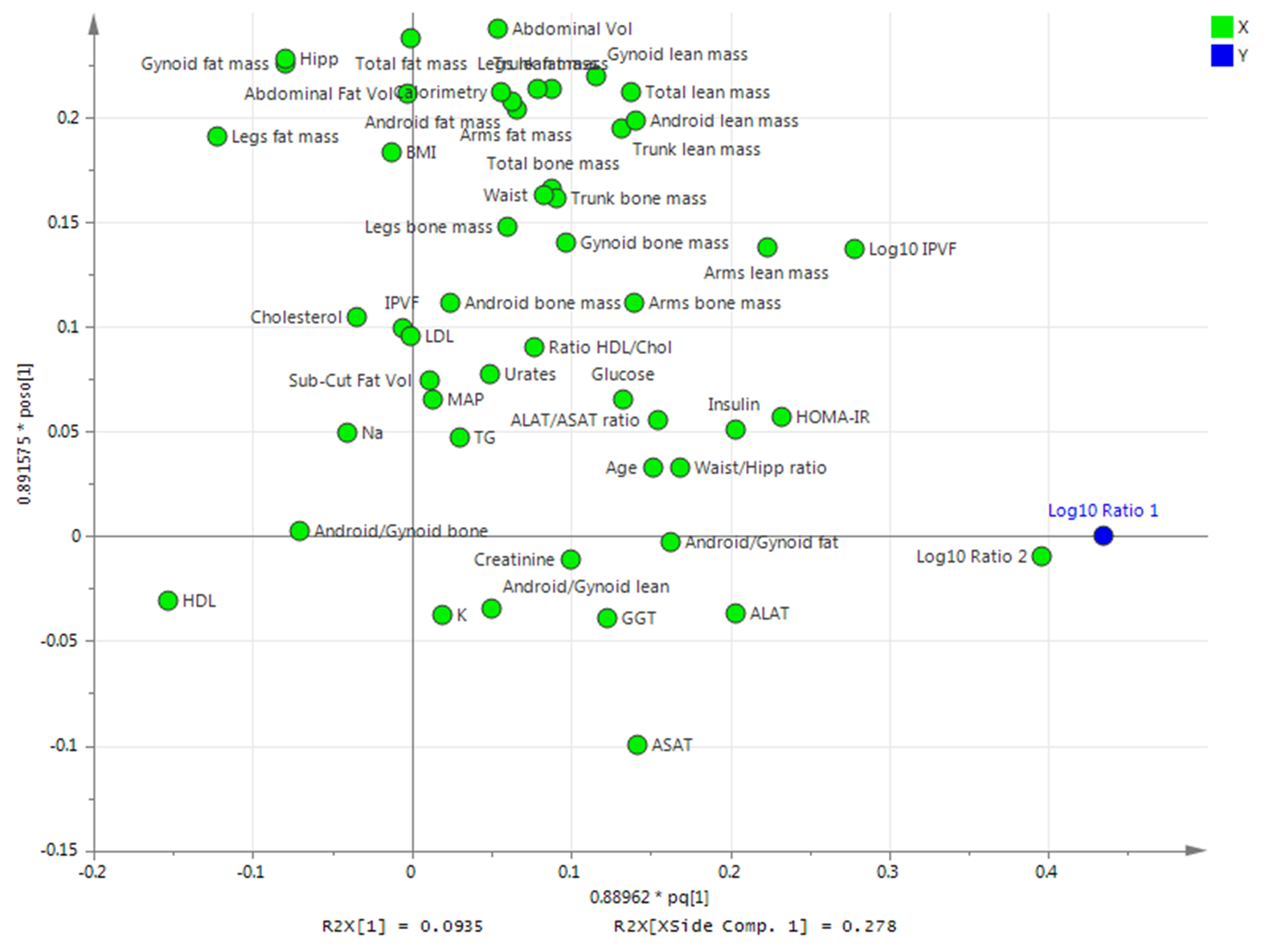

Supplement: Figure S4 — Loadings plot from orthogonal partial least square analysis of Log10 value of ratio 1 with CT and DEXA body composition parameters. OPLS model was generated with 1 predictive and 2 orthogonal components (R2X = 0.45, R2Y = 0.92, Q2Y = 0.75). (TIF) [file pone.0073445.s004.tif]

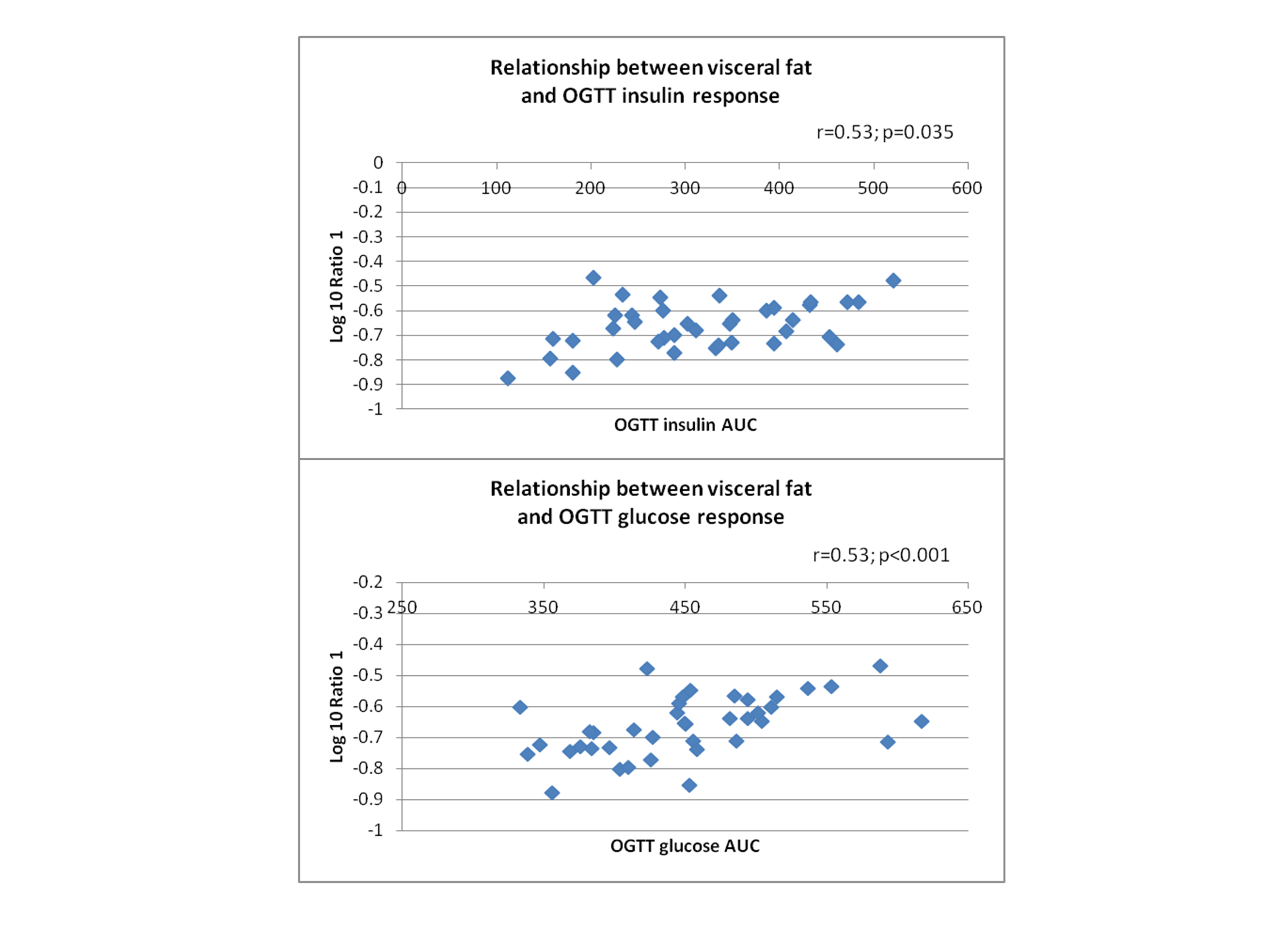

Supplement: Figure S5 — Linear regression between glucose response to OGTT and visceral adiposity. (TIF) [file pone.0073445.s005.tif]

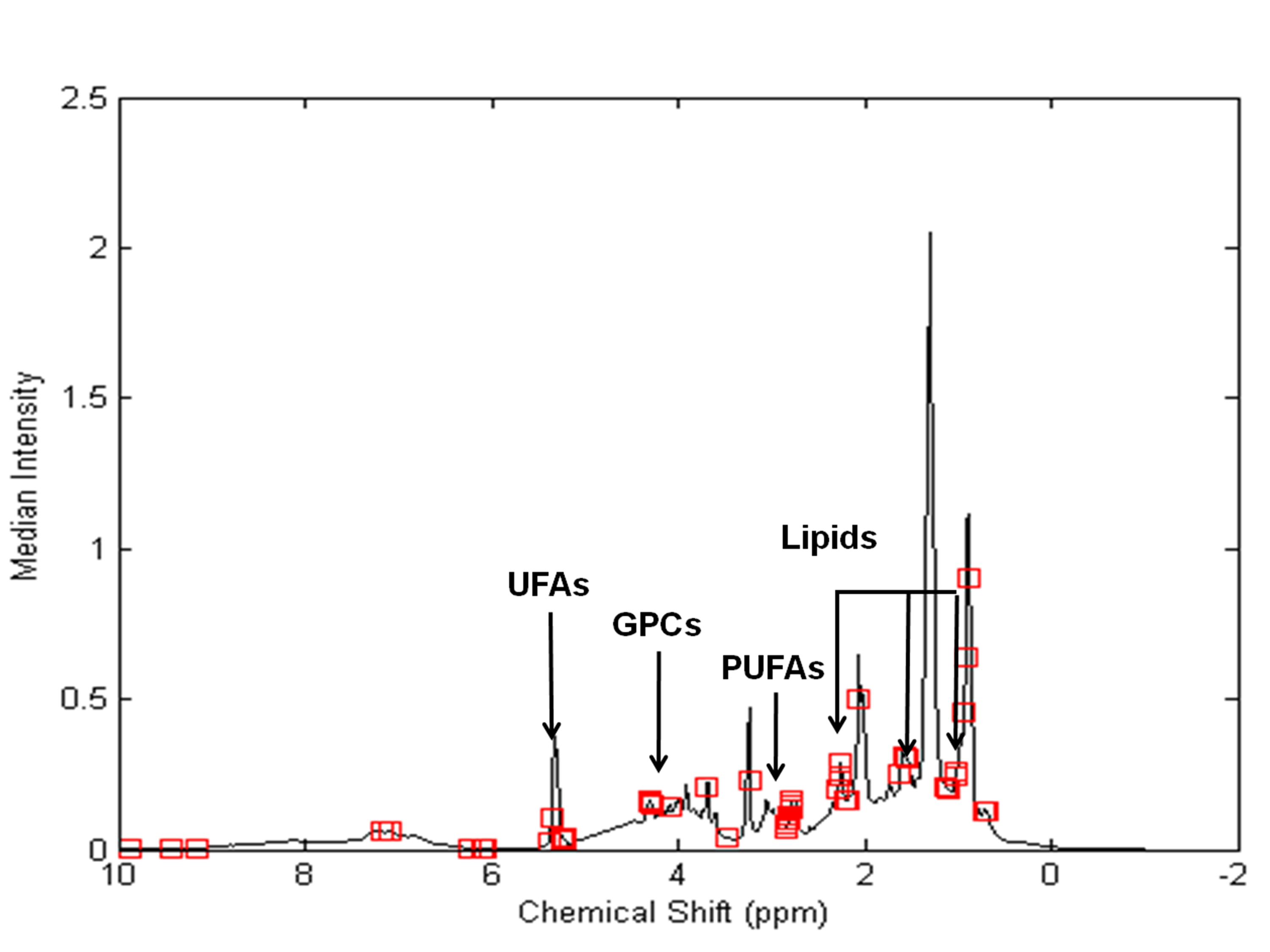

Supplement: Figure S6 — Statistical reconstruction of 1H NMR blood plasma profiles using random forest analysis to identify metabolic patterns associated with visceral adiposity (as identified with squared boxes). GPCs = glycerophospholipids, PUFAs = polyunsaturated fatty acids, UFAs = unsaturated fatty acids. (TIF) [file pone.0073445.s006.tif]

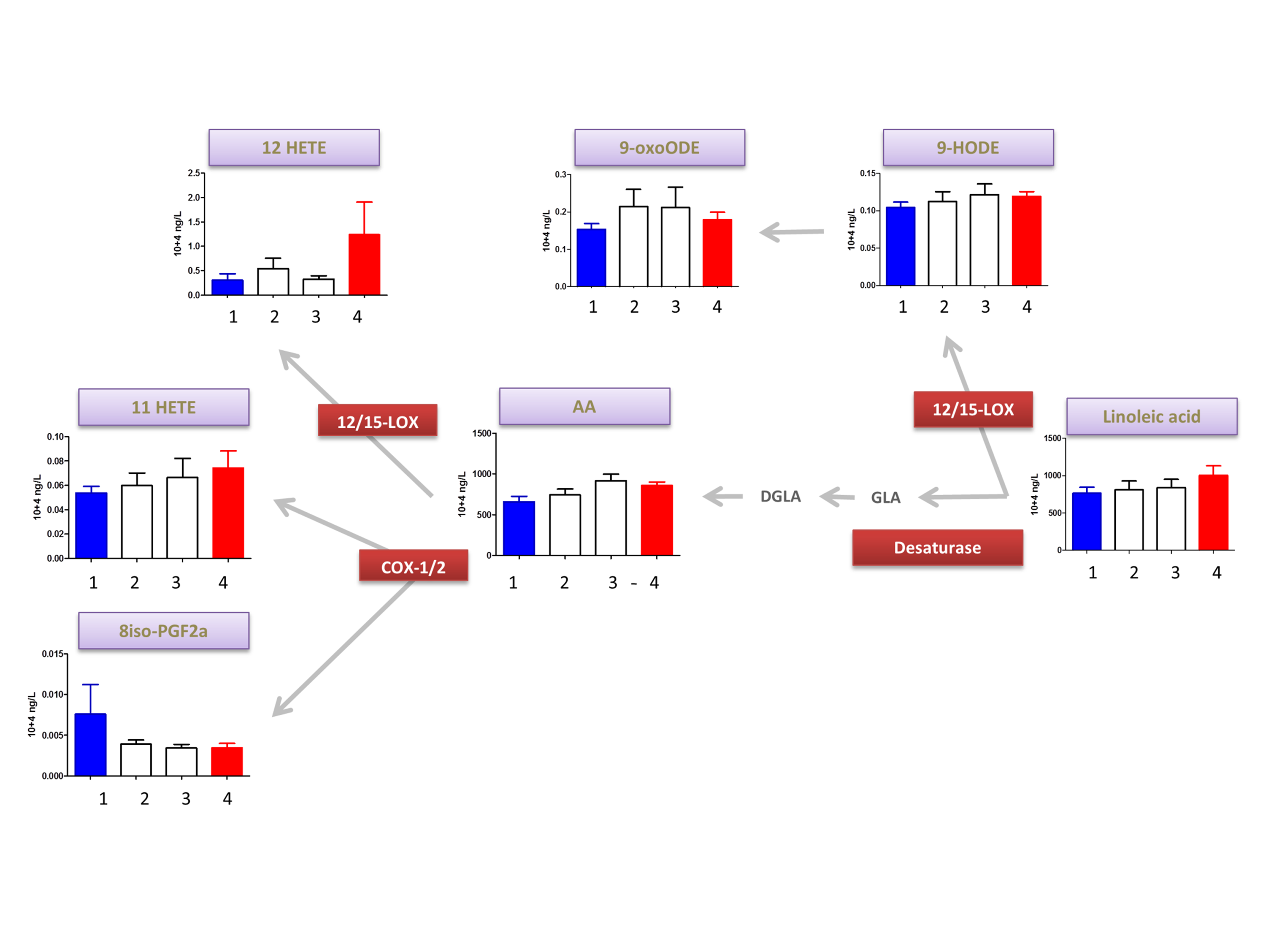

Supplement: Figure S7 — Scheme summarizing metabolic differences in the arachidonic and linoleic acid metabolic pathway across the four groups of visceral adiposity (Q1, Q2, Q3, and Q4). Bar plots describing metabolite variations in the study population stratified in four quartiles according to visceral fat adiposity (intraperitoneal fat). Statistical significance is reported in Table S2. Key: Qi: data for population quartile i according to intraperitoneal/abdominal fat ratio. 12-HETE, 12-hydroxy-eicosatetraenoic acid; 15-HETE, 12-hydroxy-eicosatetraenoic acid; 9-HODE, 9-Hydroxy-10,12-octadecadienoic acid; AA, arachidonic acid. (TIF) [file pone.0073445.s007.tif]

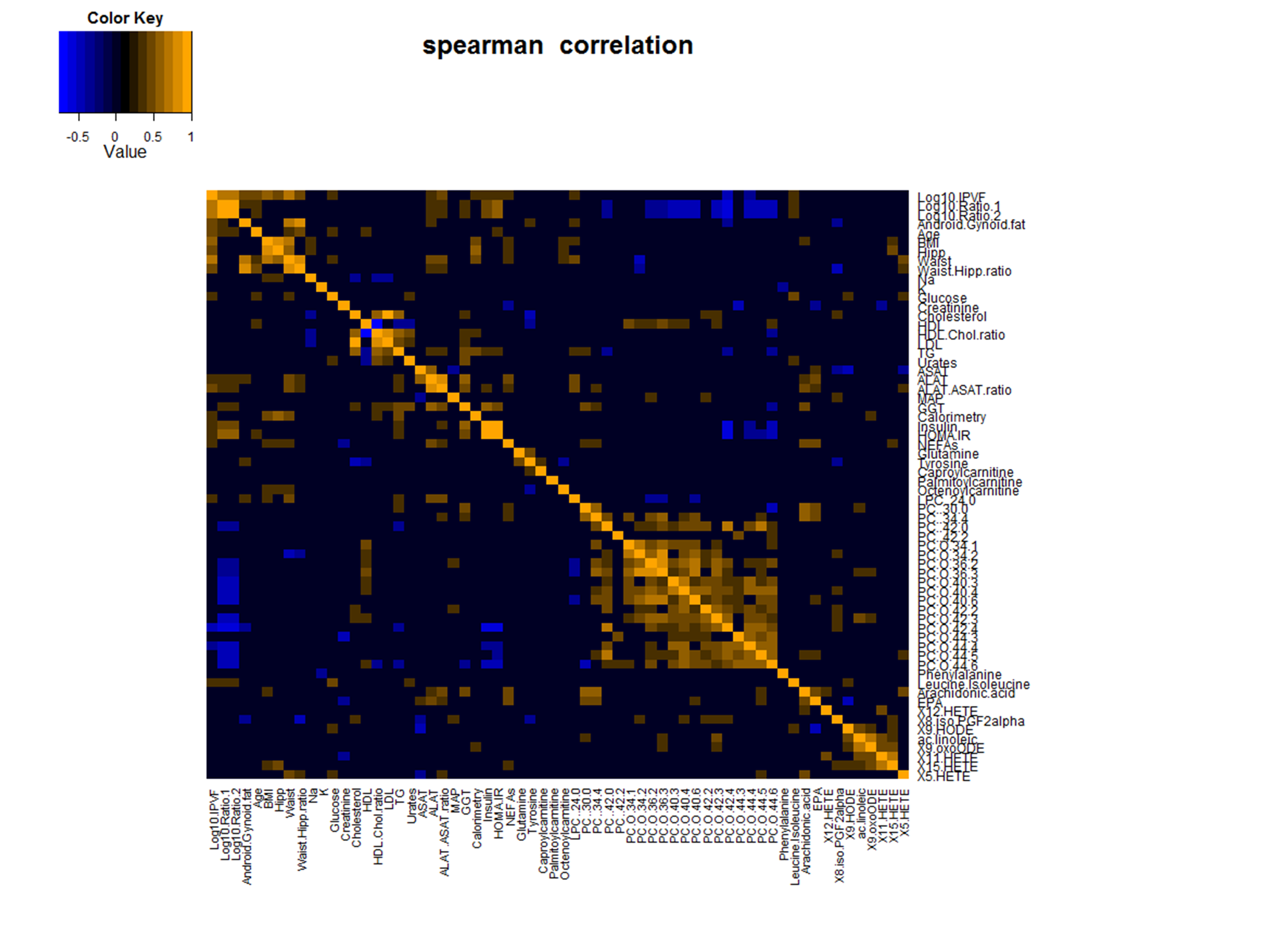

Supplement: Figure S8 — Statistically significant Spearman correlation map between visceral fat parameters CT scan, DXA data and main clinical parameters (95% confidence interval). HOMA-IR is negatively correlated with PC-O 42∶4, PC-O 44∶4, and PC-O 44∶6 lipid species, and positively with blood triglycerides, γGT, and age. ALAT/ASAT ratio showed positive correlations with AA and EPA, PC 34∶4, PC 30∶0 and LPC 24∶0 lipid species, but also fasting blood insulin, γGT and triglycerides, waist circumference and waist to hip ratio. Blue denotes negative correlation, orange denotes positive correlation, and black denotes no correlation. (TIF) [file pone.0073445.s008.tif]
