# Supplementary material for: Topographical Body Fat Distribution Links to Amino Acid and Lipid Metabolism in Healthy Non-Obese Women
Source: PLoS One. 2013 Sep 11;8(9):e73445. doi: 10.1371/journal.pone.0073445 (PMC3770640; doi:10.1371/journal.pone.0073445)
Supplement: Table S1 — Descriptive statistics of subjects stratified according to intraperitoneal fat volume. (DOCX) [file pone.0073445.s009.docx]

**Table S1: Descriptive statistics of subjects stratified according to intraperitoneal fat volume**

| **Factor** | **Q1** | **Q2** | **Q3** | **Q4** | **Mann-Whitney p value (Q1/Q4)** |
| --- | --- | --- | --- | --- | --- |
| **IPVF, mL** | **2815.8 ± 453.5** | **3937.2 ± 233.3** | **4940.6 ± 668.1** | **6112.7 ± 1045.8** | **<0.0001** |
| **Log_10_ IPVF** | **3.44±0.07** | **3.59±0.02** | **3.68±0.02** | **3.78±0.06** | **<0.0001** |
| **Waist, cm** | **93.3 ± 3.5** | **100.9 ± 9.2** | **107.2 ± 12.1** | **112.5 ± 7.66** | **0.00056** |
| **Waist/Hip ratio** | **0.8 ± 0.0** | **0.8 ± 0.1** | **0.8 ± 0.1** | **0.9 ± 0.1** | **0.00275** |
| **BMI, kg/m2** | **33.1 ± 3.4** | **34.8 ± 2.8** | **35.5 ± 3.8** | **38.5 ± 2.8** | **0.00358** |
| **Hip, cm** | **120.2 ± 5.5** | **124.6 ± 6.5** | **124.9 ± 7.5** | **129.9 ± 8.4** | **0.01506** |
| **Calorimetry, kcal/24h** | **1316 ± 130.74** | **1430 ± 151.95** | **1446 ± 170.31** | **1501 ± 207.23** | **0.01537** |
| **ALAT/ASAT ratio** | **0.78 ± 0.26** | **0.9 ± 0.17** | **1.04 ± 0.29** | **1.1 ± 0.31** | **0.03546** |
| **ALAT, U/L** | **17 ± 5.91** | **20.5 ± 6.9** | **25.9 ± 12.72** | **24.8 ± 7.96** | **0.03713** |
| **MAP, mmHg** | **54.6 ± 16.83** | **65.9 ± 19.9** | **54.7 ± 14.34** | **73.9 ± 18.11** | **0.04499** |
| HOMA-IR | 4.38 ± 2.01 | 5.1 ± 1.5 | 5.86 ± 1.71 | 6 ± 1.7 | 0.07525 |
| Insulin | 18.9 ± 9.11 | 23.1 ± 6.3 | 24.18 ± 7.07 | 24.3 ± 5.84 | 0.0892 |
| Age, years | 33.5 ± 4.8 | 32.6 ± 3.4 | 39.6 ± 4.7 | 36.6 ± 4.7 | 0.13762 |
| GGT, U/L | 18.4 ± 11.23 | 22.6 ± 10.12 | 21.33 ± 9.11 | 21.3 ± 6.77 | 0.13986 |
| Glucose, mmol/L | 5.1 ± 0.4 | 5 ± 0.29 | 5.53 ± 0.53 | 5.4 ± 0.55 | 0.19775 |
| Na, mmol/L | 140.5 ± 1.6 | 140.3 ± 1.2 | 140.4 ± 1.6 | 141.4 ± 1.2 | 0.20269 |
| K, mmol/L | 4 ± 0.2 | 4.1 ± 0.2 | 3.96 ± 0.2 | 4.1 ± 0.2 | 0.23197 |
| HDL, mmol/L | 1.53 ± 0.44 | 1.4 ± 0.24 | 1.35 ± 0.26 | 1.3 ± 0.27 | 0.23506 |
| TG, mmol/L | 1.04 ± 0.35 | 2.2 ± 2.16 | 1.55 ± 0.52 | 1.4 ± 0.51 | 0.26977 |
| Creatinine, mmol/L | 65.2 ± 9.7 | 66.9 ± 10.8 | 66.1 ± 8.2 | 68 ± 9 | 0.48613 |
| Urates, µmol/L | 266.7 ± 30.62 | 268.7 ± 51.23 | 308.8 ± 64.55 | 283.2 ± 72.22 | 0.52884 |
| HDL/Chol ratio | 3.82 ± 1.17 | 4.2 ± 0.92 | 4.15 ± 1.04 | 4.3 ± 1.14 | 0.59587 |
| Cholesterol, mmol/L | 5.49 ± 0.91 | 5.6 ± 0.94 | 5.4 ± 0.7 | 5.4 ± 0.9 | 0.70513 |
| NEFAs, µmol/L | 483.5±217.11 | 507.6±112.03 | 688.4±285.12 | 626.9±183.3 | 0.8891 |
| LDL, mmol/L | 3.48 ± 0.96 | 3.6 ± 0.85 | 3.34 ± 0.68 | 3.5 ± 0.78 | 1 |
| ASAT, U/L | 21.8 ± 2.86 | 22.1 ± 5.41 | 24.5 ± 7.79 | 22.9 ± 5.47 | 1 |

Key: BMI=body mass index, HDL-C= high density lipoprotein cholesterol, homeostasis model assessment of insulin resistance= HOMA-IR, LDL-C= low density lipoprotein cholesterol, TG= triglycerides, MAP= mean arterial blood pressure, ALAT= alanine aminotransferase, ASAT= aspartate aminotransferase, GGT= gamma-glutamyl transpeptidase, NEFAs=non esterified fatty acids.
