# Supplementary material for: Topographical Body Fat Distribution Links to Amino Acid and Lipid Metabolism in Healthy Non-Obese Women
Source: PLoS One. 2013 Sep 11;8(9):e73445. doi: 10.1371/journal.pone.0073445 (PMC3770640; doi:10.1371/journal.pone.0073445)
Supplement: Table S2 — Descriptive statistics of subjects stratified according to intraperitoneal/subcutaneous fat ratio. (DOCX) [file pone.0073445.s010.docx]

**Table S2: Descriptive statistics of subjects stratified according to intraperitoneal / subcutaneous fat ratio**

| **Factor** | **Q1** | **Q2** | **Q3** | **Q4** | **Mann-Whitney**  **p value (Q1/Q4)** |
| --- | --- | --- | --- | --- | --- |
| **Log10 Ratio 1** | **-0.78±0.05** | **-0.71±0.02** | **-0.63±0.02** | **-0.54±0.04** | **<0.0001** |
| **IPVF, mL** | **3065.1 ± 695.5** | **4223 ± 837.6** | **5005.2 ± 993.9** | **5513 ± 1561.9** | **0.00020** |
| **HOMA-IR** | **4.24 ± 2.02** | **4.95 ± 1.49** | **5.84 ± 1.63** | **6.32 ± 1.46** | **0.00893** |
| **Glucose, mmol/L** | **4.95 ± 0.35** | **5.17 ± 0.52** | **5.27 ± 0.47** | **5.51 ± 0.48** | **0.01344** |
| **Insulin** | **18.6 ± 9.21** | **22.12 ± 6.32** | **24.15 ± 7.01** | **25.63 ± 4.85** | **0.01468** |
| ALAT/ASAT ratio | 0.86 ± 0.25 | 0.91 ± 0.21 | 0.95 ± 0.3 | 1.12 ± 0.32 | 0.05354 |
| ALAT, U/L | 18.4 ± 6.11 | 19.2 ± 5.07 | 23.1 ± 8.62 | 27.5 ± 12.96 | 0.10319 |
| Age, years | 33.9 ± 4.89 | 32.8 ± 3.58 | 38 ± 4.42 | 37.6 ± 5.82 | 0.13897 |
| GGT, U/L | 20 ± 11.86 | 17.5 ± 6.88 | 20.8 ± 5.07 | 25.78 ± 10.99 | 0.17771 |
| TG, mmol/L | 1.04 ± 0.43 | 2.25 ± 2.1 | 1.35 ± 0.42 | 1.45 ± 0.62 | 0.22243 |
| HDL, mmol/L | 1.54 ± 0.43 | 1.32 ± 0.29 | 1.36 ± 0.26 | 1.34 ± 0.23 | 0.23524 |
| Creatinine, mmol/L | 65.6 ± 9.45 | 65.2 ± 11.2 | 65.6 ± 9.13 | 70 ± 6.86 | 0.28785 |
| HDL/Chol ratio | 3.77 ± 1.07 | 4.42 ± 1.22 | 4 ± 0.97 | 4.23 ± 0.96 | 0.30694 |
| Urates, µmol/L | 275.2 ± 41.93 | 263.22 ± 71.45 | 301.7 ± 76.15 | 286.7 ± 30.59 | 0.31499 |
| Na, mmol/L | 140.4 ± 1.35 | 140.8 ± 1.32 | 141.5 ± 1.58 | 139.9 ± 1.1 | 0.32894 |
| Waist/Hip ratio | 0.8 ± 0.07 | 0.81 ± 0.06 | 0.85 ± 0.07 | 0.85 ± 0.09 | 0.35104 |
| Calorimetry, kcal/24h | 1357 ± 191.78 | 1434 ± 142.61 | 1473 ± 153.05 | 1429 ± 209.57 | 0.36362 |
| Waist, cm | 97.28 ± 8.28 | 103.39 ± 8.7 | 107.83 ± 11.73 | 105.87 ± 14.1 | 0.39675 |
| Hip, cm | 122 ± 5.47 | 128 ± 7.48 | 127.12 ± 6.42 | 122.5 ± 9.69 | 0.62631 |
| ASAT, U/L | 21.4 ± 3.24 | 21.4 ± 4.48 | 24.4 ± 6.6 | 24.1 ± 7.05 | 0.64728 |
| MAP, mmHg | 57.8 ± 18.6 | 71.1 ± 19.75 | 56.5 ± 15.57 | 63.7 ± 19.51 | 0.64953 |
| NEFAs, µmol/L | 544.5±201.51 | 580.6±301.38 | 594.4±186.88 | 586.9±187.63 | 0.64963 |
| LDL, mmol/L | 3.5 ± 0.97 | 3.56 ± 0.88 | 3.27 ± 0.67 | 3.54 ± 0.73 | 0.93958 |
| Cholesterol, mmol/L | 5.52 ± 1.01 | 5.58 ± 0.85 | 5.25 ± 0.73 | 5.54 ± 0.91 | 0.93967 |
| BMI , kg/m2 | 34.01 ± 3.27 | 36.34 ± 3.62 | 36.98 ± 2.97 | 34.61 ± 4.42 | 0.93969 |
| K, mmol/L | 4.05 ± 0.18 | 4.1 ± 0.18 | 3.98 ± 0.25 | 4.05 ± 0.18 | 1 |

Key: BMI=body mass index, HDL-C= high density lipoprotein cholesterol, homeostasis model assessment of insulin resistance= HOMA-IR, LDL-C= low density lipoprotein cholesterol, TG= triglycerides, MAP= mean arterial blood pressure, ALAT= alanine aminotransferase, ASAT= aspartate aminotransferase, GGT= gamma-glutamyl transpeptidase, NEFAs=non esterified fatty acids.
