# Supplementary material for: Topographical Body Fat Distribution Links to Amino Acid and Lipid Metabolism in Healthy Non-Obese Women
Source: PLoS One. 2013 Sep 11;8(9):e73445. doi: 10.1371/journal.pone.0073445 (PMC3770640; doi:10.1371/journal.pone.0073445)
Supplement: Table S3 — Metabolite variations across of subjects stratified according to intraperitoneal fat volume. (DOCX) [file pone.0073445.s011.docx]

**Table S3: Metabolite variations across of subjects stratified according to intraperitoneal fat volume**

| **Metabolites (concentration)** | **Q1** | **Q2** | **Q3** | **Q4** | **Mann-Whitney p value (Q1/Q4)** |
| --- | --- | --- | --- | --- | --- |
| **Tyrosine, µmol/L** | **68.71 ± 17.75** | **69.2 ± 20.04** | **67.67 ± 16.89** | **94.5 ± 20.14** | **0.00567** |
| **Palmitoylcarnitine, µmol/L** | **0.06 ± 0.02** | **0.1 ± 0.02** | **0.07 ± 0.02** | **0.1 ± 0.03** | **0.00567** |
| **PC-O 44:4, µmol/L** | **0.85 ± 0.3** | **0.7 ± 0.19** | **0.58 ± 0.24** | **0.5 ± 0.14** | **0.00567** |
| **PC-O 42:4, µmol/L** | **1.27 ± 0.29** | **1.2 ± 0.35** | **1.03 ± 0.41** | **0.9 ± 0.24** | **0.01991** |
| **LPC 24:0, µmol/L** | **0.25 ± 0.1** | **0.5 ± 0.21** | **0.54 ± 0.37** | **0.5 ± 0.32** | **0.03034** |
| **PC 42:2, µmol/L** | **0.22 ± 0.07** | **0.2 ± 0.09** | **0.16 ± 0.08** | **0.2 ± 0.07** | **0.03498** |
| AA, ng/100 µL | 657.67 ± 205.96 | 743.6 ± 231.74 | 916.5 ± 256.52 | 859 ± 130.74 | 0.06027 |
| 12-HETE, ng/100 µL | 0.31 ± 0.39 | 0.5 ± 0.67 | 0.33 ± 0.22 | 1.2 ± 2.12 | 0.07889 |
| PC-O 44:3, µmol/L | 0.21 ± 0.07 | 0.2 ± 0.04 | 0.17 ± 0.05 | 0.2 ± 0.05 | 0.10201 |
| Caproylcarnitine, µmol/L | 0.19 ± 0.11 | 0.2 ± 0.04 | 0.16 ± 0.08 | 0.3 ± 0.19 | 0.1333 |
| PC-O 44:6, µmol/L | 1.47 ± 0.59 | 1.2 ± 0.36 | 1.08 ± 0.45 | 1.2 ± 0.37 | 0.15286 |
| PC-O 34:2, µmol/L | 11.49 ± 3.41 | 8.9 ± 2.51 | 8.12 ± 1.84 | 9.7 ± 4.75 | 0.1564 |
| PC-O 40:3, µmol/L | 1.37 ± 0.24 | 1.4 ± 0.27 | 1.07 ± 0.55 | 1.1 ± 0.45 | 0.17733 |
| Glutamine, µmol/L | 662.67 ± 133.7 | 658.8 ± 108.76 | 739.3 ± 164.38 | 813.6 ± 264.22 | 0.18231 |
| PC-O 40:6, µmol/L | 3.68 ± 1.1 | 3 ± 0.97 | 2.86 ± 1.1 | 3 ± 0.94 | 0.21102 |
| PC-O 44:5, µmol/L | 2.17 ± 0.77 | 1.9 ± 0.66 | 2.06 ± 0.68 | 1.8 ± 0.7 | 0.22046 |
| PC 34:4, µmol/L | 1.11 ± 0.35 | 1.4 ± 0.51 | 1.8 ± 1.06 | 1.5 ± 0.78 | 0.2428 |
| 9-HODE, ng/100 µL | 0.1 ± 0.02 | 0.1 ± 0.04 | 0.12 ± 0.05 | 0.1 ± 0.02 | 0.24561 |
| 8-iso-PGF2α, ng/100 µL | 0.008 ± 0.011 | 0.004 ± 0.002 | 0.003 ± 0.001 | 0.003 ± 0.002 | 0.2695 |
| PC-O 34:1, µmol/L | 9.85 ± 2.39 | 8.7 ± 2.64 | 9.34 ± 3.02 | 8.8 ± 2.19 | 0.26971 |
| PC 42:0, µmol/L | 0.56 ± 0.21 | 0.6 ± 0.21 | 0.49 ± 0.09 | 0.4 ± 0.14 | 0.26992 |
| Leu+ILe, µmol/L | 193.56 ± 47.65 | 204.3 ± 57.82 | 216.9 ± 44.72 | 213.3 ± 34.19 | 0.27751 |
| PC-O 36:3, µmol/L | 7.19 ± 1.61 | 6.6 ± 2.24 | 6.19 ± 1.69 | 6.4 ± 2.3 | 0.27751 |
| Octenoylcarnitine, µmol/L | 0.04 ± 0.02 | 0 ± 0.02 | 0.05 ± 0.03 | 0.1 ± 0.04 | 0.307 |
| 15-HETE, ng/100 µL | 0.06 ± 0.05 | 0.1 ± 0.18 | 0.14 ± 0.3 | 0.1 ± 0.13 | 0.30722 |
| PC-O 42:3, µmol/L | 0.89 ± 0.19 | 0.9 ± 0.19 | 0.78 ± 0.37 | 0.8 ± 0.21 | 0.40018 |
| PC-O 36:2, µmol/L | 11.79 ± 2.77 | 10.5 ± 2.3 | 9.39 ± 3.17 | 11.1 ± 2.52 | 0.62405 |
| PC-O 40:4, µmol/L | 2.65 ± 0.61 | 2.7 ± 0.79 | 2.33 ± 0.74 | 2.5 ± 0.94 | 0.66072 |
| PC 30:0, µmol/L | 4.18 ± 0.62 | 4.9 ± 1.74 | 6.65 ± 1.71 | 5.2 ± 2.4 | 0.71969 |
| Phenylalanine, µmol/L | 52.97 ± 13.67 | 52.1 ± 6.82 | 60.49 ± 22.46 | 54.3 ± 12.88 | 0.74386 |
| PC-O 42:2, µmol/L | 0.56 ± 0.22 | 0.6 ± 0.19 | 0.48 ± 0.17 | 0.5 ± 0.16 | 0.78018 |

NB: Blood plasma metabolites highlighted by multivariate analyses are reported as mean values ± SD. Key: Qi: data for population quartile i according to intraperitoneal / abdominal fat ratio. 12-HETE, 12-hydroxy-eicosatetraenoic acid; 15-HETE, 12-hydroxy-eicosatetraenoic acid; 9-HODE, 9-Hydroxy-10,12-octadecadienoic acid; AA, arachidonic acid; LPC, Lysophosphatidylcholines; PC, Phosphatidylcholines; PC-O, 1-O-alkyl-2- acylglycerophosphocholines; SM, Sphingomyelines; SM-OH, Hydroxy-Sphingomyelin.
