# Supplementary material for: Topographical Body Fat Distribution Links to Amino Acid and Lipid Metabolism in Healthy Non-Obese Women
Source: PLoS One. 2013 Sep 11;8(9):e73445. doi: 10.1371/journal.pone.0073445 (PMC3770640; doi:10.1371/journal.pone.0073445)
Supplement: Table S4 — Metabolic variations across subjects stratified according to intraperitoneal/subcutaneous fat ratio. (DOCX) [file pone.0073445.s012.docx]

**Table S4: Metabolic variations across subjects stratified according to intraperitoneal / subcutaneous fat ratio**

| **Metabolites (concentration)** | **Q1** | **Q2** | **Q3** | **Q4** | **Mann-Whitney**  **p value (Q1/Q4)** |
| --- | --- | --- | --- | --- | --- |
| **PC-O 42:4, µmol/L** | **1.34 ± 0.33** | **1.09 ± 0.28** | **1.08 ± 0.38** | **0.83 ± 0.22** | **0.00298** |
| **PC-O 40:3, µmol/L** | **1.41 ± 0.27** | **1.46 ± 0.38** | **1.21 ± 0.32** | **0.91 ± 0.46** | **0.01417** |
| **PC-O 44:4, µmol/L** | **0.8 ± 0.3** | **0.67 ± 0.24** | **0.62 ± 0.19** | **0.51 ± 0.17** | **0.01721** |
| **PC-O 44:6, µmol/L** | **1.52 ± 0.56** | **1.22 ± 0.3** | **1.09 ± 0.5** | **1.05 ± 0.32** | **0.01721** |
| **Glutamine, µmol/L** | **615.56 ± 107.95** | **748 ± 193.49** | **729.6 ± 164.3** | **776.5 ± 227.88** | **0.01991** |
| **Phenylalanine, µmol/L** | **49.9 ± 14.16** | **50.47 ± 8.45** | **60.19 ± 22.24** | **59.05 ± 9.43** | **0.02218** |
| **Leu+Ile, µmol/L** | **181.44 ± 53.02** | **214.2 ± 56.71** | **200.2 ± 25.48** | **231 ± 33.25** | **0.03498** |
| **8-iso-PGF2α, ng/100 µL** | **0.008 ± 0.011** | **0.004 ± 0.001** | **0.003 ± 0.002** | **0.003 ± 0.001** | **0.037** |
| **PC-O 44:5, µmol/L** | **2.29 ± 0.74** | **2.03 ± 0.55** | **1.91 ± 0.73** | **1.7 ± 0.71** | **0.04113** |
| **PC-O 40:6, µmol/L** | **3.81 ± 0.86** | **3.27 ± 1.09** | **2.87 ± 0.92** | **2.67 ± 1.01** | **0.04347** |
| PC 42:0, µmol/L | 0.65 ± 0.23 | 0.48 ± 0.16 | 0.47 ± 0.07 | 0.47 ± 0.14 | 0.05347 |
| PC-O 42:2, µmol/L | 0.66 ± 0.23 | 0.56 ± 0.14 | 0.52 ± 0.15 | 0.46 ± 0.18 | 0.05347 |
| Tyrosine, µmol/L | 61.97 ± 11.02 | 80.54 ± 22.21 | 72.52 ± 15.14 | 84.38 ± 28.16 | 0.05347 |
| PC-O 40:4, µmol/L | 2.79 ± 0.56 | 2.9 ± 0.73 | 2.38 ± 0.66 | 2.12 ± 0.89 | 0.06027 |
| 9-HODE, ng/100 µL | 0.1 ± 0.02 | 0.12 ± 0.05 | 0.1 ± 0.02 | 0.13 ± 0.04 | 0.08606 |
| PC-O 42:3, µmol/L | 0.89 ± 0.2 | 0.92 ± 0.14 | 0.87 ± 0.3 | 0.66 ± 0.26 | 0.09471 |
| PC-O 36:3, µmol/L | 7.04 ± 1.68 | 6.7 ± 2.61 | 6.73 ± 1.75 | 5.87 ± 1.67 | 0.1333 |
| PC-O 44:3, µmol/L | 0.21 ± 0.06 | 0.2 ± 0.04 | 0.15 ± 0.06 | 0.16 ± 0.05 | 0.1333 |
| PC-O 36:2, µmol/L | 11.29 ± 2.64 | 11.86 ± 2.68 | 10.07 ± 2.8 | 9.48 ± 2.58 | 0.15232 |
| PC 42:2, µmol/L | 0.2 ± 0.06 | 0.19 ± 0.11 | 0.15 ± 0.07 | 0.15 ± 0.07 | 0.1564 |
| 15-HETE, ng/100 µL | 0.08 ± 0.05 | 0.17 ± 0.29 | 0.1 ± 0.13 | 0.1 ± 0.18 | 0.18231 |
| Palmitoylcarnitine, µmol/L | 0.07 ± 0.02 | 0.07 ± 0.03 | 0.08 ± 0.02 | 0.1 ± 0.04 | 0.18231 |
| PC-O 34:1, µmol/L | 9.94 ± 2.22 | 9.78 ± 3.84 | 8.42 ± 2.11 | 8.59 ± 1.07 | 0.20546 |
| LPC 24:0, µmol/L | 0.36 ± 0.25 | 0.51 ± 0.24 | 0.52 ± 0.36 | 0.46 ± 0.31 | 0.21613 |
| PC 30:0, µmol/L | 4.43 ± 1.48 | 5.17 ± 2.35 | 6.1 ± 1.82 | 5.23 ± 1.81 | 0.35623 |
| PC-O 34:2, µmol/L | 10.66 ± 3.5 | 9.31 ± 3.51 | 8.89 ± 4.36 | 9.26 ± 2.26 | 0.35623 |
| PC 34:4, µmol/L | 1.3 ± 0.46 | 1.53 ± 1.14 | 1.42 ± 0.52 | 1.54 ± 0.75 | 0.40018 |
| Octenoylcarnitine, µmol/L | 0.04 ± 0.02 | 0.05 ± 0.02 | 0.06 ± 0.04 | 0.05 ± 0.02 | 0.56728 |
| Caproylcarnitine, µmol/L | 0.22 ± 0.1 | 0.2 ± 0.09 | 0.15 ± 0.07 | 0.29 ± 0.19 | 0.60378 |
| 12-HETE, ng/100 µL | 0.6 ± 0.72 | 0.28 ± 0.3 | 1.14 ± 2.15 | 0.42 ± 0.35 | 1 |
| AA, ng/100 µL | 784.22 ± 236.64 | 764.9 ± 314.46 | 839 ± 209.8 | 801.3 ± 145.13 | 1 |

NB: Blood plasma metabolites highlighted by multivariate analyses are reported as mean values ± SD. Key: Qi: data for population quartile i according to intraperitoneal / abdominal fat ratio. 12-HETE, 12-hydroxy-eicosatetraenoic acid; 15-HETE, 12-hydroxy-eicosatetraenoic acid; 9-HODE, 9-Hydroxy-10,12-octadecadienoic acid; AA, arachidonic acid; LPC, Lysophosphatidylcholines; PC, Phosphatidylcholines; PC-O, 1-O-alkyl-2- acylglycerophosphocholines; SM, Sphingomyelines; SM-OH, Hydroxy-Sphingomyelin.
