# Supplementary material for: Topographical Body Fat Distribution Links to Amino Acid and Lipid Metabolism in Healthy Non-Obese Women
Source: PLoS One. 2013 Sep 11;8(9):e73445. doi: 10.1371/journal.pone.0073445 (PMC3770640; doi:10.1371/journal.pone.0073445)
Supplement: Table S5 — Metabolite VIP ranking based on PLS models generated at V2+V0. (DOCX) [file pone.0073445.s013.docx]

**Table S5: Metabolite VIP ranking based on PLS models generated at V2+V0.**

| **Metabolites** | **VIP value from PLS on Log_10_ Ratio2** | **VIP value from PLS on Log_10_ Ratio1** | **VIP value from PLS**  **on Log_10_ IPVF** |
| --- | --- | --- | --- |
| PC-O 42:4 | 1.62463 | 1.63222 | 1.60008 |
| PC-O 44:4 | 1.55424 | 1.5943 | 1.76521 |
| PC-O 40:3 | 1.5024 | 1.44428 | 0.866988 |
| PC-O 44:6 | 1.25399 | 1.2672 | 0.847983 |
| PC 42:0 | 1.25241 | 1.29157 | 0.884864 |
| PC-O 40:6 | 1.22028 | 1.19217 | 0.648336 |
| PC-O 42:3 | 1.21006 | 1.18702 | 0.636001 |
| Palmitoylcarnitine | 1.12717 | 1.05693 | 1.48413 |
| PC-O 40:4 | 1.12599 | 1.07166 | 0.364103 |
| PC-O 42:2 | 1.06802 | 1.05542 | 0.643008 |
| PC-O 44:3 | 1.01355 | 1.08636 | 1.16068 |
| Leu+Ile | 1.01123 | 1.0269 | 1.00639 |
| PC-O 44:5 | 0.986982 | 1.04186 | 0.382152 |
| PC-O 36:2 | 0.959983 | 0.878381 | 0.729142 |
| PC-O 36:3 | 0.881786 | 0.802766 | 0.84928 |
| Tyrosine | 0.867216 | 0.948231 | 1.68241 |
| PC-O 34:1 | 0.803788 | 0.783285 | 0.630124 |
| PC 42:2 | 0.703113 | 0.721358 | 0.498745 |
| PC-O 34:2 | 0.678934 | 0.622151 | 1.00986 |
| PC 30:0 | 0.499468 | 0.45276 | 1.09922 |
| Glutamine | 0.467011 | 0.630062 | 1.01396 |
| Phenylalanine | 0.413564 | 0.469868 | 0.477011 |
| Caproylcarnitine | 0.35506 | 0.438269 | 0.870764 |
| Octenoylcarnitine | 0.261832 | 0.221735 | 0.903488 |
| PC 34:4 | 0.0675216 | 0.0645886 | 1.09681 |

**NB:** PLS models were generated using 1 predictive component based on data generated at V0 and V2.
