# Supplementary material for: Topographical Body Fat Distribution Links to Amino Acid and Lipid Metabolism in Healthy Non-Obese Women
Source: PLoS One. 2013 Sep 11;8(9):e73445. doi: 10.1371/journal.pone.0073445 (PMC3770640; doi:10.1371/journal.pone.0073445)
Supplement: Text S1 — Sample Preparation and 1H-NMR Spectroscopic Analysis. (DOCX) [file pone.0073445.s014.docx]

**Text S1: Sample preparation for Biocrates Life Sciences Absolute*IDQ*^TM^ kit analysis**

The Biocrates Life Sciences Absolute*IDQ*^TM^ kit was used for EDTA plasma samples as previously published [1]. Well plate preparation and sample application and extraction were carried out according to the manufacturer's instructions. A final volume of 10 μl of plasma was loaded onto the provided 96-well plate, containing isotopically labeled internal standards. Liquid chromatography was realized on a Dionex Ultimate 3000 ultra high pressure liquid chromatography (UHPLC) system (Dionex AG, Olten, Switzerland) coupled to a 3200 Q TRAP mass spectrometer (AB Sciex; Foster City, CA, USA) fitted with a TurboV ion source operating in electrospray ionization (ESI) mode. Sample extracts (20 µl) were injected two times (in positive and negative ESI modes) via direct infusion using a gradient flow rate of 0-2.4 min: 30 µl/min, 2.4-2.8 min: 200 µl/min, 2.9-3 min: 30 µl/min. MS source parameters were set at: desolvation temperature (TEM): 200°C, high voltage: -4500 V (ESI -), 5500 V (ESI +), curtain (CUR) and nebuliser (GS1 and GS2) gases: nitrogen; 20, 40, and 50 psi; respectively, nitrogen collision gas pressure: 5 mTorr. MS/MS acquisition was realised in scheduled reaction monitoring (SRM) mode with optimised declustering potential values for the 163 metabolites screened in the assay. Raw data files (Analyst software, version 1.5.1; AB Sciex, Foster City, CA, USA) were imported into the provided analysis software MetIQ to calculate metabolite concentrations. List of all detectable metabolites is available from Biocrates Life Sciences, Austria (<http://biocrates.com>). Individual lipid species were annotated as follows: [lipid class] [total number of carbon atoms]:[total number of double bonds]. For example, PC 34:4 reflects a phosphatidylcholine species comprising 34 carbon atoms and 4 double bonds. Lipid classes were labelled as follow: LPC, Lysophosphatidylcholines; PC, Phosphatidylcholines; PC-*O*, 1-O-alkyl-2-acylglycerophosphocholines; SM, Sphingomyelines; and SM-*OH*, Hydroxy-Sphingomyelin.

Reference List

1. Römisch-Margl W, Prehn C, Bogumil R, Röhring C, Suhre K et al. (2011) Procedure for tissue sample preparation and metabolite extraction for high-throughput targeted metabonomics. Metabonomics .
