# Supplementary material for: Topographical Body Fat Distribution Links to Amino Acid and Lipid Metabolism in Healthy Non-Obese Women
Source: PLoS One. 2013 Sep 11;8(9):e73445. doi: 10.1371/journal.pone.0073445 (PMC3770640; doi:10.1371/journal.pone.0073445)
Supplement: Text S2 — Sample preparation for Biocrates Life Sciences Absolute IDQ ™ kit analysis. (DOCX) [file pone.0073445.s015.docx]

### Text S2: Sample preparation and inflammation markers quantification by UPLC-ESI-MS/MS using isotope dilution technique

Based on previously published work (Baur et al., 2011; Deems et al., 2007) a method to measure a panel of 63 inflammatory markers was developed in house. 5 µl of the internal standard solution (0.1 ng/µl) is added to 100µL of plasma sample.10 µl of butylated hydroxytoluene (0.359 µM) is added and the mixture is acidified by adding 15 µl of citric acid (1N). A volume of 550 µl of methanol/ethanol (1:1, v:v) was added and samples were mixed during 15 min at 4° C before being centrifuged (500 g, 10 min, 4°C). The organic phase was evaporated to dryness under constant nitrogen flow and the residues were solubilised with 80 µl water, followed by the addition of 20 µL of acetonitrile, before being centrifuged at 3500 rpm for 1 min at 4° C. The supernatant was transferred into LC-MS vials before analysis. Analyses were carried out by liquid chromatography coupled to tandem mass spectrometry (LC-MS/MS). LC was realized on a Dionex Ultimate 3000 ultra pressure liquid chromatography (UPLC) system (Dionex AG, Olten, Switzerland). MS detection was realized on a 5500 Q TRAP mass spectrometer (AB Sciex; Foster City, CA, USA) operating in ESI negative ionization mode. Gradient chromatographic separation was performed on an Acquity BEH C18 column (2.1 x 150 mm, 1.7 µm; Waters, Milford, USA). The injection volume was 5 µl and the column was maintained at 50 °C. The mobile phase consisted of water containing 1% acetic acid (eluent A) and acetonitrile (eluent B) at a constant flow rate set at 450 µl/min. Gradient elution started from 20% B with a linear increase to 50% B at 6 min, from 50% to 95% B at 13 min, hold for 3 min at 95% B, before going back to 20% B at 16.1 min and re-equilibration of the column for an additional 11 min. Analytes were monitored in the scheduled selected reaction monitoring (scheduled SRM) mode provided within the Analyst software (version 1.5.1; AB Sciex, Foster City, CA, USA). The SRM detection window time was set at 120 sec with a target scan time of 0.5 sec. Nitrogen was used as curtain and desolvation gas at the respective pressure of CUR: 20, GS1: 70, GS2: 20 (arbitrary unit). Block source temperature was maintained at 600°C, with the respective voltages: ISV: -4000 V, EP: -10 V, CXP: -5 V. A 15-points calibration curve was realized prior to sample analysis by measuring different dilutions of the standard solution (0-10 ng) fortified with an isotopically labeled internal standard (ISs) mix. Data processing was realized using Analyst software (version 1.5.1; AB Sciex, Foster City, CA, USA). Peak area ratio of each analyte versus its corresponding internal standard or surrogate marker was calculated.
