# Supplementary material for: Topographical Body Fat Distribution Links to Amino Acid and Lipid Metabolism in Healthy Non-Obese Women
Source: PLoS One. 2013 Sep 11;8(9):e73445. doi: 10.1371/journal.pone.0073445 (PMC3770640; doi:10.1371/journal.pone.0073445)
Supplement: Text S3 — Sample preparation and inflammation markers quantification by UPLC-ESI-MS/MS using isotope dilution technique. (DOCX) [file pone.0073445.s016.docx]

### Text S3: Sample Preparation and ^1^H-NMR Spectroscopic Analysis

Heparin blood plasma samples (400 µL) were introduced into 5 mm NMR tubes with 200 µL of deuterated phosphate buffer solution (KH_2_PO_4_ with a final concentration of 0.2M). Deuterium was employed as locking substance. 24 hours urine samples (400 µL) were introduced into 5 mm NMR tubes with 200 µL of deuterated phosphate buffer solution (KH_2_PO_4_ with a final concentration of 0.2M, and containing 1 mM of sodium 3-(trimethylsilyl)-[2,2,3,3-2H_4_]-1-propionate (TSP). Metabolic profiles were measured on a Bruker Avance III 600 MHz spectrometer equipped with an inverse 5mm cryogenic probe at 300 K (Bruker Biospin, Rheinstetten, Germany). Urine samples were measured using a standard pulse sequence with water suppression during a relaxation delay (RD) of 4 s. Standard ^1^H-NMR one-dimensional pulse sequence with water suppression (RD=4s), Carr-Purcell-Meiboom-Gill (CPMG) spin-echo sequence with water suppression (RD=4s), and diffusion-edited sequence (RD=1s) where acquired for each plasma sample. For each one dimensional experiment 16 and 32 scans were collected using 98 K data points for urine and plasma samples, respectively. ^1^H-NMR spectra were processed using TOPSPIN (version 2.1, Bruker, Germany) software package prior to Fourier transformation. The acquired NMR spectra were manually phased and baseline corrected, and referenced to the chemical shift of the anomeric proton of α-glucose at δ 5.236 for plasma spectra and of TSP at δ 0.00 for urine. The assignment of the ^1^H-NMR resonances to specific metabolites was achieved by matching our in-house developed NMR database of pure compounds and using literature data [1,2]. Metabolite identification was confirmed by 2D ^1^H-^1^H COrrelation SpectroscopY (COSY) [3], ^1^H-^1^H TOtal Correlation SpectroscopY (TOCSY) [4] and ^1^H-^13^C Heteronuclear Single Quantum Correlation (HSQC) [5] NMR techniques.

Reference List

1. Fan TW (1996) Metabolite profiling by one- and two-dimensional NMR analysis of complex mixtures. Progress in Nuclear Magnetic Resonance Spectroscopy 28: 161-219.

2. Nicholson JK, Foxall PJ, Spraul M, Farrant RD, Lindon JC (1995) 750 MHZ ^1^H and ^1^H-^13^C NMR spectroscopy of human blood plasma. Anal Chem 67: 793-811.

3. Hurd RE (1990) Gradient-Enhanced Spectroscopy. J Magn Reson 87: 422-428.

4. Bax A, Davis D (1985) MLEV-17-Based Two-Dimensional Homonuclear Magnetization Transfer Spectroscopy. J Magn Reson 65: 355-360.

5. Bodenhausen G, Ruben DJ (1980) Natural abundance nitrogen-15 NMR by enhanced heteronuclear spectroscopy. Chemical Physics Letters 69: 185-189.
